# Scoping Review of Economic Analyses of Rare Kidney Diseases

**DOI:** 10.1016/j.ekir.2024.09.004

**Published:** 2024-09-12

**Authors:** Blake Angell, Siyuan Wang, Thomas Gadsden, Monica Moorthy, Charu Malik, Jonathan Barratt, Olivier Devuyst, Ifeoma I. Ulasi, Daniel P. Gale, Agnivo Sengupta, Anna Palagyi, Vivekanand Jha, Stephen Jan

**Affiliations:** 1 The George Institute for Global Health, University of New South Wales, Sydney, Australia; 2 International Society of Nephrology, Brussels, Belgium; 3 Department of Cardiovascular Sciences, University of Leicester, Leicester, UK; 4 Department of Physiology, Mechanisms of Inherited Kidney Disorders, University of Zurich, Zurich, Switzerland; 5 Division of Nephrology, Cliniques Universitaires Saint-Luc, UCLouvain, Brussels, Belgium; 6 Renal Unit, Department of Medicine, College of Medicine, University of Nigeria, Ituku-Ozalla, Enugu, Nigeria; 7 Renal Unit, Department of Medicine, University of Nigeria Teaching Hospital, Ituku-Ozalla, Enugu, Nigeria; 8 Renal Unit, Department of Internal Medicine, Alex Ekwueme Federal University Teaching Hospital, Abakaliki, Nigeria; 9 National Registry of Rare Kidney Diseases, Bristol, UK; 10 Department of Renal Medicine, University College London, London, UK; 11 The George Institute for Global Health, University of New South Wales, New Delhi, India; 12 School of Public Health, Imperial College, London, UK; 13 Prasanna School of Public Health, Manipal Academy of Higher Education, Manipal, India

**Keywords:** cost-effectiveness, economic burden, rare kidney disease

## Abstract

**Introduction:**

Rare kidney diseases (RKDs) place a substantial economic burden on patients and health systems, the extent of which is unknown and may be systematically underestimated by health economic techniques. We aimed to investigate the economic burden and cost-effectiveness evidence base for RKDs.

**Methods:**

We conducted a systematic scoping review to identify economic evaluations, health technology assessments, and cost-of-illness studies relating to RKDs, published since 2012.

**Results:**

A total of 161 published studies, including 66 cost-of-illness studies and 95 economic evaluations; 72 grey literature reports were also included. Most published literature originated from high-income nations, particularly the USA (81 studies), and focused on a handful of diseases, notably renal cell carcinomas (70) and systemic lupus erythematosus (36). Limited evidence was identified from lower-income settings and there were few studies of genetic conditions, which make up most RKDs. Some studies demonstrated the cost-effectiveness of existing treatments; however, there were limited considerations of broader economic impacts on patients that may be important to those with RKDs. Included health technology assessments highlighted difficulties in obtaining high-quality clinical evidence for treatments in very small patient populations, and often considered equity issues and other patient impacts qualitatively alongside clinical and economic evidence in their recommendations.

**Conclusion:**

We found large gaps in the economic evidence base for RKDs and limited adaptation of methods to account for the uniqueness of these diseases. There may be significant scope for innovation in building an investment case for RKD treatments, as well as in decision-making processes to inform investment decisions.

## Graphical abstract

**Figure:**
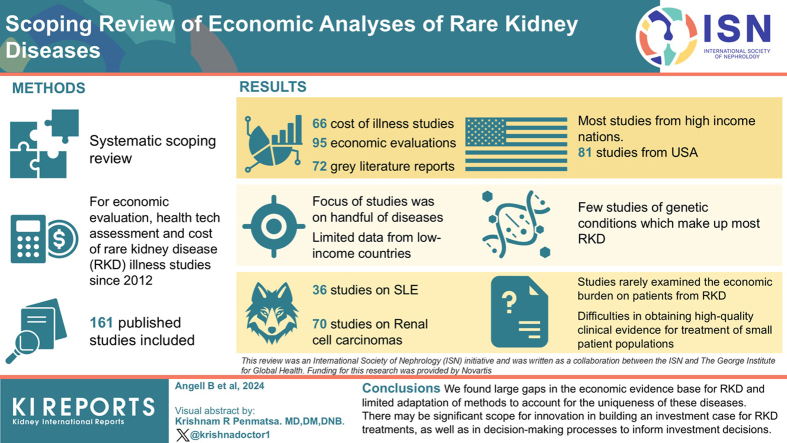

Although there is no universally accepted definition, rare kidney diseases (RKDs) are often defined as a group of over 150 conditions affecting the kidneys, many of which are inherited, with a prevalence of about 60 to 80 cases per 100,000 in Europe and the USA.^1^, ^2^, ^3^ Less is known about the prevalence of these conditions in the rest of the world, especially low- and lower-middle-income countries.

The diagnosis and management of RKDs present distinct challenges to patients, their families, and health systems. Patients and their families face prolonged periods of multiple tests and uncertainty before diagnosis, while the diseases themselves contribute significantly to morbidity, premature mortality, and economic stress for those affected.^2^^,^^3^ For health systems, small numbers of patients, unidentified causes, lack of diagnostic tools, limited treatment options and complex care needs challenge the ability of even well-resourced health systems to provide appropriate and responsive care.^2^^,^^4^ Further, though each individual disease is uncommon, collectively, RKDs place significant burdens on health systems.^4^ Emerging data from the UK have demonstrated that people with RKDs have a higher likelihood of experiencing kidney failure but are less than half as likely to die with chronic kidney disease stages 3 to 5, therefore accounting for a disproportionate share of patients on kidney replacement therapy. This suggests that better treatments for these diseases at earlier stages could effectively curb the growing demand for expensive kidney replacement therapy and yield disproportionate economic benefits across health systems.^5^

Investing in treatments and care for RKD necessitates trade-offs. Despite the life-changing potential of effective therapies for patients and their families, their overall impact on population health may be limited by small numbers of patient for individual diseases. Therefore, investing scarce resources in high-cost treatments for relatively small groups seemingly detracts from potentially larger population health gains from alternative investments in more common public health problems. This is further complicated by a large unmet need, because effective targeted treatments are available for less than 10% of RKDs. Incentives to the pharmaceutical industry to develop treatments for RKDs may be weak, given the small potential market, unless countered by specific regulatory frameworks to encourage investment. Further, given the overrepresentation of people with RKD among those receiving kidney replacement therapy,^5^ such conditions pose major financial challenges for low- and middle-income countries, which are seeing a higher proportion of their health care budgets being used for kidney replacement therapy (relative to high-income settings).^4^^,^^6^ Given the substantial burden of health care costs experienced by individual patients, much of which is borne out of pocket in low- and middle-income nations, there may be strong equity arguments for regulatory provisions that address the barriers new RKD treatments encounter in conventional HTA processes. Approaches to funding treatments for rare diseases vary across countries.^7^^,^^8^ Some nations, such as Latvia and Bulgaria, exempt treatments for rare diseases from HTA processes, whereas others, including the UK, Sweden, and Australia, have developed guidelines for drugs for rare diseases that go beyond usual cost-effectiveness considerations. Others, such as Slovakia and Romania, do not have explicit processes for rare diseases. Policy development is often limited by the lack of large databases.

We conducted a systematic scoping review of the published and grey literature to investigate the current economic evidence base supporting investment into treatments for RKDs. We sought to identify the evidence base estimating the economic impact of RKDs on health systems around the world as well as the comparative cost-effectiveness of available treatments for these conditions. A scoping review method was used because we sought to capture diverse studies incorporating different patient groups, methods, and health systems, as well as identifying gaps in the existing literature. Although no previous reviews were found investigating the economic burden of RKDs, several scoping reviews have examined the cost of illness studies in rare diseases or have focused specifically on economic evaluations of some rare diseases (not limited to those affecting the kidneys) in certain contexts.^9^, ^10^, ^11^ One recently published study examined economic evaluations of inherited rare diseases in a selected group of high-income nations, finding that patient costs are rarely included in such studies, which the authors suggest may be systematically underestimating the cost-effectiveness of such treatments.^11^ Here, we build on these previous studies to draw together the global economic evidence base, including economic evaluations, health technology assessments, and cost-of-illness studies in the field of RKDs. In doing so, we hope to illustrate the current case for investment in treatments for RKDs, identify shortcomings in the health economic approaches used in these studies, and identify potential areas for improvement.

## Methods

We conducted a scoping review to identify economic evaluations, cost analyses, and health technology assessments of interventions for RKDs. The review specifically sought to identify the following:

1. the economic burden of RKDs and relative cost-effectiveness of available treatment;
2. the extent and type of economic evidence available to inform investment decisions for treatments of RKDs in countries around the world;
3. gaps in the existing literature relating to the income classification of countries where evidence exists, types of treatment and diseases covered, the applicability of methods and perspectives taken in these analyses, and;
4. whether any specific adjustments to HTA processes had been identified that had occurred or were recommended within this evidence base.

### Study Selection

Studies published between December 2012 and the time of search (May 2023) were included if they examined the cost, cost-effectiveness, or value of treatment for a rare kidney disease. The list of eligible RKDs was developed based on the existing Orphanet list of rare diseases and European Rare Kidney Disease registry.^12^^,^^13^ A brief literature review was conducted to confirm that all conditions were associated with kidney disease. Conditions without an association with kidney disease were not included in this study. Some conditions, such as renal cell carcinomas, which are not necessarily rare, were only eligible for inclusion if they were associated with rare syndromes. The full list of conditions is provided in the Supplementary Material.

Searches were conducted in the PubMed, EMBASE, and Global Health databases. Briefly, searches contained terms (keywords and MESH subject headings) to identify relevant methodologies (e.g., economic evaluations, health technology assessments, budget impact analyses, cost-of-illness studies, and other cost analyses) and eligible RKDs. No restrictions were placed on the country of study or interventions included. The search strategy used in Medline is provided in the Supplementary Table S1. This was supplemented by a search of the grey literature involving targeted searches of the websites of HTA institutions and national or regional kidney association websites, as well as Google searches of key terms to identify non peer-reviewed and publicly available studies. Websites searched included specific HTA institutions, including the UK National Institute for Health and Care Excellence, Australia’s Medical Services Advisory Committee and Pharmaceutical Benefits Advisory Committee, and the Canadian Agency for Drugs and Technologies in Health. Key kidney and rare disease-related sources were also searched, including the International Rare Diseases Research Consortium, The UK Kidney Association, Overton, and Kidney Disease Improving Global Outcomes Controversies Conferences.^14^, ^15^, ^16^, ^17^, ^18^

### Data Extraction and Synthesis

Results from the database searches were uploaded into Rayyan,^19^ and titles and abstracts were screened by 1 of 3 authors (SW, TG, or BA) for potential suitability for inclusion in this review. Full texts were then screened for inclusion by the same authors who proceeded to extract data from the included studies. Discrepancies were resolved by consensus in all circumstances. Data were extracted on study characteristics and findings, including country, method, time horizon, discount rate and perspective of analysis, the disease or diseases of interest, types of costs included, intervention studied, results, key drivers of costs, whether budget impacts were considered or analyzed, and if the paper raised issues or shortcomings with HTA or funding systems about RKD treatments. We synthesized the evidence in accordance with method of Arksey and O’Malley^20^ (2005) and the Preferred Reporting Items for Scoping Reviews and Meta-Analyses Extension for Scoping Reviews guidance^21^ (Supplementary Material**)**.

## Results

### Overview of Peer-Reviewed Economic Evidence Base

A total of 1802 articles were identified from the database search after the removal of duplicates and screened for potential inclusion (Supplementary Figure S1). Full texts were screened of 260 studies and ultimately, 161 peer-reviewed published papers met our inclusion criteria for this review: 66 cost-of-illness studies^22^, ^23^, ^24^, ^25^, ^26^, ^27^, ^28^, ^29^, ^30^, ^31^, ^32^, ^33^, ^34^, ^35^, ^36^, ^37^, ^38^, ^39^, ^40^, ^41^, ^42^, ^43^, ^44^, ^45^, ^46^, ^47^, ^48^, ^49^, ^50^, ^51^, ^52^, ^53^, ^54^, ^55^, ^56^, ^57^, ^58^, ^59^, ^60^, ^61^, ^62^, ^63^, ^64^, ^65^, ^66^, ^67^, ^68^, ^69^, ^70^, ^71^, ^72^, ^73^, ^74^, ^75^, ^76^, ^77^, ^78^, ^79^, ^80^, ^81^, ^82^, ^83^, ^84^, ^85^, ^86^, ^87^ and 95 economic evaluations,^88^, ^89^, ^90^, ^91^, ^92^, ^93^, ^94^, ^95^, ^96^, ^97^, ^98^, ^99^, ^100^, ^101^, ^102^, ^103^, ^104^, ^105^, ^106^, ^107^, ^108^, ^109^, ^110^, ^111^, ^112^, ^113^, ^114^, ^115^, ^116^, ^117^, ^118^, ^119^, ^120^, ^121^, ^122^, ^123^, ^124^, ^125^, ^126^, ^127^, ^128^, ^129^, ^130^, ^131^, ^132^, ^133^, ^134^, ^135^, ^136^, ^137^, ^138^, ^139^, ^140^, ^141^, ^142^, ^143^, ^144^, ^145^, ^146^, ^147^, ^148^, ^149^, ^150^, ^151^, ^152^, ^153^, ^154^, ^155^ including 27 cost analyses.^156^, ^157^, ^158^, ^159^, ^160^, ^161^, ^162^, ^163^, ^164^, ^165^, ^166^, ^167^, ^168^, ^169^, ^170^, ^171^, ^172^, ^173^, ^174^, ^175^, ^176^, ^177^, ^178^, ^179^, ^180^, ^181^, ^182^ The characteristics of these studies are shown in Table 1. Most of the economic evidence emanated from the United States (78 studies) and other high-income nations, with limited evidence from low- and middle-income nations (17 studies including 10 from China). The vast majority of studies (128) considered costs from the health care payer perspective. Studies examining 30 different RKDs were included in our review, though most focused on renal cell carcinoma (71 studies) and systemic lupus erythematosus (36). Very few studies explicitly noted shortcomings in current health technology assessment processes regarding RKDs. Approximately half of the included studies examined the economic burden of the RKD on patients and health systems without examining the impact of an intervention, whereas the remainder were economic evaluations of interventions to treat RKDs.

**Table 1:** General characteristics of included studies

Characteristics	Total *n* (%)	Cost-of-illness (*n* = 66)	Cost-effectiveness analyses (*n* = 68)	Cost analyses (*n* = 27)
Year of publication (*n* = 161)				
2012–2017	80 (50)	26	47	7
2018–2023	81 (50)	40	21	20
Country of study^a^ (*n* = 169)				
High-income	150 (89)	66	61	23
USA	81 (48)	32	33	16
Europe	38 (22)	20	12	6
UK	10 (6)	2	7	1
Other high income	21 (12)	12	9	0
Low or middle income	19 (11)	4	10	5
Other low or middle income	9 (5)	1	4	4
China	10 (6)	3	6	1
Disease studied (*n* = 161)				
Renal cell carcinoma	70 (43)	10	47	13
Systemic lupus erythematosus	36 (22)	27	5	4
Systemic sclerosis	8 (5)	7	0	1
Thrombotic thrombocytopenic purpura	7 (4)	1	2	3
Tuberous sclerosis complex	7 (4)	6	1	0
Autosomal dominant polycystic kidney disease	6 (4)	2	4	0
Amyloid light chain amyloidosis	4 (2)	3	0	1
Acute intermittent porphyria	3 (2)	2	0	1
Gaucher disease type 1	3 (2)	1	1	1
Alpha-1 antitrypsin deficiency	2 (1)	2	0	0
Eosinophilic granulomatosis with polyangiitis	2 (1)	1	1	0
Fabry disease	2 (1)	0	2	0
Neurofibromatosis type 1	2 (1)	1	0	1
Other^b^	9 (6)	2	5	2
Perspective adopted^a^ (*n* = 162)				
Health system or health payer	128 (79)	51	50	27
Societal	14 (9)	7	7	0
Other (e.g., patient or unclear)	20 (12)	8	12	0

a Multiple perspectives and countries covered in some studies.

b Including acute hepatic porphyria, familial mediterranean fever, galactosemia, giant cell arteritis, idiopathic nephrotic syndrome, polyarteritis nodosa, thrombotic microangiopathy, X-linked hypophosphatemia, and sickle cell anemia.

### The Economic Burden of RKDs (Cost-of-Illness Studies)

In total, 66 cost-of-illness studies examined RKDs (Table 1 and Supplementary Table S2). The majority of these were conducted in high-income nations, particularly the USA (*n* = 32). Seventeen RKDs were covered across the cost-of-illness studies; 26 studies focused on systemic lupus erythematosus (including complications such as nephritis), 10 on renal cell carcinoma (including metastatic), 7 on systemic sclerosis, and 6 on tuberous sclerosis complex. Sample sizes for included studies ranged from 47 patients with tuberous sclerosis complex,^74^ to a nation-wide study of 22,258 systemic lupus erythematosus cases in Taiwan.^34^

Nearly all studies were retrospective, using insurance databases or patient registries to collect cost data. Only 2 studies prospectively recruited patients,^32^^,^^35^^,^ whereas 4 collected patient costs through audits of clinical records and 2 estimated costs using economic models.^24^^,^^62^ Most studies adopted a health system or payer perspective (*n* = 51), 8 took a patient perspective, and 7 took a societal perspective.^22^, ^23^, ^24^^,^^35^^,^^52^^,^^55^^,^^58^ Of the latter, 5 studies calculated indirect costs using the human capital approach,^22^^,^^23^^,^^35^^,^^52^^,^^58^ whereas Knarborg *et al.*^55^ (2022) estimated indirect costs “as the differences in earning between matched cases and controls based on earned income and various social security compensation,” and Connolly *et al.*^24^ (2021) modeled the differences in lifetime earnings for patients with acute hepatic porphyria compared to the general population.^24^^,^^55^

The cost of illness studies demonstrated a large economic burden associated with RKDs, though most reported on diseases that involve multiple systems. Despite variation across specific conditions and health systems, studies identified that patients with RKDs incurred higher costs than those without. Across all included studies, regardless of disease type, direct medical costs were the main cost drivers, including hospitalization, utilization of outpatient services, and medication costs. In studies that adopted a societal perspective, key cost drivers included medical costs and foregone earnings related to long-term disability and sick leave.

### Economic Evaluations of Treatments for RKDs

Sixty-eight studies examined the cost-effectiveness or cost utility of interventions for treatments of rare kidney diseases, including 2 cost-benefit analyses. An additional 27 studies were cost analyses that compared the relevant costs of treating people living with RKDs with specific treatments compared to usual care. The majority of cost-effectiveness and cost-utility studies were based in high-income countries, again predominantly the USA (*n* = 30), compared to low-and-middle income settings (10 studies, including 6 in China) (Table 2 and Supplementary Table S3). Most of these studies (*n* = 44) investigated the cost-effectiveness of first line and second-line treatments for renal cell carcinomas in different settings. Nearly all studies (*n* = 61) were modelled economic evaluations, with a few retrospective cohort studies (*n* = 7) that assessed cost-effectiveness. The time horizon of studies ranged from a few days to lifetime depending on the condition of interest. The most common discount rate used was 3% (*n* = 36) and most studies (*n* = 50) took the health system perspective, assessing the direct medical costs and effectiveness of treatments on national health care sector and third-party payers. Some studies also took a broader societal perspective, incorporating indirect costs such as loss in productivity and increased caregiver burden into the evaluation process (*n* = 7).^95^^,^^96^^,^^131^^,^^133^^,^^135^^,^^145^^,^^180^ Although diverse, the majority of studies examined drug-based treatments (*n* = 54), particularly for the first line and second-line treatments for renal cell carcinomas (*n* = 43). Aside from pharmaceutical treatments, included studies also evaluated the cost-effectiveness of diagnostic tests, screening and prevention programs, and disease management programs.

**Table 2:** Summary of economic evaluation evidence

Characteristics of economic evaluations included	Total *n* (%)	Cost-effective (*n* = 42)	Not cost-effective (*n* = 16)	Mixed results (*n* = 10)
Country of study^a^ (*n* = 71)				
High-income	61 (86)	38	13	10
USA	33 (46)	19	7	7
Europe	12 (17)	8	3	1
UK	7 (10)	5	0	2
Other high income	9 (13)	6	3	0
Low or middle income	10 (14)	9	1	0
Other low or middle income	4 (6)	3	1	0
China	6 (8)	6	0	0
Intervention type (*n* = 68)				
Pharmaceutical interventions	53 (78)	31	15	7
Nonpharmaceutical interventions	15 (22)	11	1	3
Intervention studied^a^ (*n* = 73)				
Renal cell carcinoma				
First line interventions	33 (45)	19	12	2
Pazopanib vs. sunitinib	6 (8)	5	1	0
Pembrolizumab plus axitinib vs. sunitinib	4 (5)	1	2	1
Nivolumab based strategies vs. sunitinib^b^	11 (15)	7	3	1
Others vs. sunitinib^c^	8 (11)	3	5	0
Other first line interventions^d^	4 (5)	3	1	0
Second line interventions^e^	11 (15)	8	2	1
Screening programs and other	9 (12)	5	3	1
Systemic lupus erythematosus				
Belimumab vs. soc	3 (4)	2	0	1
Lupus self-management program	1 (1)	1	0	0
Autosomal dominant polycystic kidney disease				
Screening programs	2 (3)	2	0	0
Other^f^	2 (3)	1	1	0
Other disease interventions^g^	12 (16)	7	2	3

Soc, AAA.

a Multiple interventions and countries covered in some studies.

b Including nivolumab plus ipilimumab and nivolumab plus cabozantinib.

c Including lenvatinib plus pembrolizumab, anlotinib, and avelumab plus axitinib.

d Including nivolumab plus ipilimumab vs. pembrolizumab plus axitinib, and nivolumab plus ipilimumab vs. pazopanib.

e Including everolimus vs. sorafenib, cabozantinib vs. everolimus, nivolumab vs. everolimus, ofaxitinib vs. sorafenib, nivolumab vs. axitinib, pazopanib, everolimus and cabozantinib and cabozantinib vs. axitinib, and cabozantinib vs. nivolumab.

f Including tolvaptan vs. soc and ace (angiotensin-converting enzymes inhibitors) vs. arb (angiotensin ii receptor blockers).

g Including interventions for eosinophilic granulomatosis with polyangiitis, galactosemia, Gaucher disease type 1, giant cell arteritis, idiopathic nephrotic syndrome, thrombotic microangiopathy, tuberous sclerosis complex, and X-linked hypophosphatemia.

### Cost-Effectiveness of Included Interventions

Of the 68 studies evaluating the cost-effectiveness of treatments, 42 provided favorable cost-effectiveness evidence in support of the intervention, 16 indicated that the intervention was not cost-effective, and the remaining 10 reported mixed results. Of the 53 studies evaluating pharmaceutical interventions, 15 reported unfavorable cost-effectiveness results, whereas 12 out of 15 studies evaluating nonpharmaceutical interventions were found to be cost-effective.^139^^,^^140^^,^^143^, ^144^, ^145^, ^146^^,^^148^^,^^150^, ^151^, ^152^, ^153^ Details of the cost-effectiveness found for different treatment comparisons are shown in Table 2.

Most studies identified medication costs as a key cost driver, consequently impacting cost-effectiveness outcomes.^89^, ^90^, ^91^, ^92^, ^93^, ^94^, ^95^^,^^98^^,^^100^^,^^101^^,^^104^^,^^105^^,^^118^ Other key cost drivers included adverse event management, disease management, administration, and terminal care costs. For screening programs, the key drivers of costs also included screening costs, assessment costs, administrative costs, as well as the cost of early treatment of screened patients and the cost of delayed treatment of unscreened patients. These were generally outweighed by the benefits of reduced downstream health care costs. For studies taking a broader societal perspective, productivity losses and caregiver burden were also important cost components. Those studies that adopted a societal perspective highlighted the increased cost-effectiveness if broader costs were considered. Bindra *et al.*,^135^ for example, assessed the cost-effectiveness of Acthar gel (respiratory corticotropin injection) verses standard-of-care treatments in moderate-to-severe systemic lupus erythematosus and found that taking the broader societal perspective as opposed to the payer perspective lowered the Incremental Cost Effectiveness Ratio from $133,110 per quality-adjusted life year to $70,827 per quality-adjusted life year.^135^

### Cost and Cost-Minimization Analyses

Twenty-seven studies were cost and cost consequences analyses, of which the majority (*n* = 24) were cost minimization studies estimating the cost impacts of introducing new diagnostics or treatments for RKDs compared to the current standard of care (Table 2 and Supplementary Table S3). Like the other included literature, most studies were conducted in the USA (*n* = 16) and focused on a handful of diseases. Most adopted a health system perspective and identified potential cost savings (*n* = 23) for health systems, predominantly due to reductions in drug acquisition costs.

### Grey Literature

A total of 72 documents were identified in the grey literature,^183^, ^184^, ^185^, ^186^, ^187^, ^188^, ^189^, ^190^, ^191^, ^192^, ^193^, ^194^, ^195^, ^196^, ^197^, ^198^, ^199^, ^200^, ^201^ 65 of which were health technology reports from the UK, Australia, and Canada (Table 3). Forty-eight of these recommended funding for interventions, 17 recommended not supporting estimate and 6 were inclusive, including 1 grey literature report on the cost of tuberous sclerosis complex in the USA. Once again, most reports focused on interventions to treat renal cell carcinoma (31 reports). Most reports focused on treatments for conditions; however, the introduction of certain diagnostic and screening interventions for some RKDs was considered cost-effective in some contexts.^183^^,^^193^^,^^194^

**Table 3:** Summary of the included grey literature

Characteristics of grey literature reports included	Total *n* (%)	Recommended with conditions (*n* = 48)	Not recommended (*n* = 17)	Inconclusive evidence^a^ (*n* = 6)
Country of study^b^ (*N* = 72)				
UK	32 (44)	25	4	3
AU	15 (21)	9	5	0
Canada	20 (28)	12	8	0
Other	5 (7)	2	0	3
Intervention studied^b^ (*N* = 72)				
Renal cell carcinoma				
Avelumab-with-axitinib	2 (3)	1	1	0
Axitinib	2 (3)	2	0	0
Cabozantinib based strategies^c^	5 (7)	5	0	0
Lenvatinib based strategies^d^	4 (6)	3	1	0
Nivolumab based strategies^e^	4 (6)	4	0	0
Pembrolizumab based strategies^f^	5 (7)	4	1	0
Sorafenib	2 (3)	1	1	0
Sunitinib	2 (3)	1	1	0
Other^g^	5 (7)	4	1	0
Systemic lupus erythematosus				
Belimumab	3 (4)	1	2	0
Anifrolumab	2 (3)	1	1	0
Atypical hemolytic uremic syndrome				
Eculizumab	2 (3)	1	1	0
Ravulizumab	4 (6)	4	0	0
Autosomal dominant polycystic kidney disease				
Tolvaptan	3 (4)	2	1	0
Fabry disease				
Agalsidase alfa	1 (1)	0	1	0
Migalastat	3 (4)	3	0	0
Pegunigalsidase	1 (1)	1	0	0
Enzyme replacement therapies	1 (1)	0	0	1
Other diseases studied^h^	21 (29)	12	4	5

a Includes 1 cost of illness study identified in the grey literature.

b Multiple interventions and countries covered in some studies.

c Including cabozantinib and cabozantinib plus nivolumab.

d Including lenvatinib, lenvatinib plus everolimus, and Lenvatinib plus pembrolizumab.

e Including nivolumab and nivolumab-with-ipilimumab.

f Including pembrolizumab, pembrolizumab plus axitinib, and pembrolizumab plus lenvatinib.

g Including tivozanib, vinflunine, pazopanib, everolimus and bevacizumab (first line), sorafenib (first- and second line), sunitinib (second-line) and temsirolimus (first-line).

h Including daratumumab for AL amyloidosis, C3 glomerulopathy, selumetinib for neurofibromatosis type 1, caplacizumab for acquired thrombotic thrombocytopenic purpura, screening programs for genetic diseases, enzyme replacement therapy for lysosmal storage diseases, enzyme replacement therapies for mucopolysaccharidosis type 1, rituximab for antineutrophil cytoplasmic antibody (ANCA)-associated vasculitis, ciclosporin for idiopathic steroid-resistant nephrotic syndrome, cost-of-illness of tuberous sclerosis complex, and burosumab for X-linked hypophosphatemia.

Grey literature reports commonly raised issues around inadequate clinical or economic evidence to establish the value of a treatment. Nonetheless, most health technology assessments supported funding and often deemed the treatment cost-effective. In addition to cost-effectiveness, included health technology assessments considered other criteria to inform investment decisions, including safety, clinical effectiveness, equity, access and rule of rescue, community preference, and social benefits. Although likely important to formulating an investment case for treatments, these benefits were generally presented qualitatively as part of supporting evidence alongside clinical and economic data. Safety and clinical effectiveness were the 2 most important criteria considered by all assessments in their decision-making process. Safety was incorporated through both the severity of adverse events, as well as the cost of managing these adverse events as part of the economic evaluation process. For clinical effectiveness, assessments reported the clinical effectiveness of the proposed treatments through the literature reviews of relevant clinical trials and synthesizing effectiveness data (noting the limitations in data due to small patient numbers described above). Six assessments explicitly considered equity within their decision criteria. Two documents considered the broader social benefits of the proposed treatments, including the ability to contribute to society or continue education, cost savings from personal expenses for patients and carers for transportation and housing, and caregivers’ ability to return to work, leading to increased productivity.^184^^,^^189^

## Discussion

This review has provided a comprehensive assessment of the published economic literature on RKDs. Although the evidence base is diverse and somewhat fragmented, our findings highlight that the economic burden of rare kidney disease treatments on patients, health care systems, and society is substantial. Direct medical costs facing patients are often large and generally increased with severity of the disease, the existence of multiple conditions, hospital admission, and medication costs. Some treatments were identified as cost-effective in certain contexts in this review as were some diagnostic and screening interventions for RKDs. This suggests that well-targeted interventions can offer cost-effective improvements in patient outcomes, even under traditional measures of cost-effectiveness, despite relatively small target populations and indicates the potential for early detection and management.

Dialysis as a downstream consequence of advanced kidney disease is exceedingly expensive, albeit generally still considered cost-effective and reimbursed in many middle- and high-income countries. The review suggests that early and effective treatment interventions could potentially reduce the reliance on such expensive therapies, offering the promise of both health and economic benefits. However, the complexity of RKDs, which often affect multiple organ systems, complicates the assessment of their economic impact. Separating the specific impact of these conditions on the kidneys from their effects on other organ systems remains challenging, because these diseases often present with systemic manifestations contributing to their overall burden. This complexity necessitates a comprehensive approach to economic evaluation that considers the multifaceted nature of these conditions.

The evidence-base pulled together through this review focused primarily on several key conditions and came from a handful of countries. Over half of the cost-effective evidence found relates to renal cell carcinomas, a heterogeneous group of tumors (≥40 subtypes) with distinct genetic, histologic, and phenotypic characteristics. Although individually rare, together they constitute 85% of all kidney cancers.^202^ Further, most cost-of-illness studies considered diseases involving multiple systems, such as systemic lupus erythematosus, with few studies focusing on genetic conditions comprising most RKDs.

Among the published academic literature, we found limited evidence of methodologies specifically adapted to account for the unique challenges posed by rare diseases to health systems and policymakers. As a result, the literature may underestimate the actual societal economic burden of RKDs. For instance, from a societal perspective, the economic burden of 379 rare diseases in the USA was estimated to reach US $1 trillion in 2019, less than half of which was attributed to direct medical costs.^203^ Similar evidence should be developed for kidney diseases, and it would be crucial to develop an accurate and compelling argument for policymakers to invest in preventing and treating RKDs.

Other, less tangible impacts, such as health system strengthening impacts, were also largely overlooked in included studies. Previous work has investigated how investing in rare disease programs can build health system resilience by fostering multidisciplinary expertise and improved care quality,^204^^,^^205^ enabling faster decision-making and coordinated response to emerging health care challenges. This is particularly relevant for low- and middle-income countries that have few centers of excellence for RKDs. Research into rare diseases can also spill over into broader gains in medical knowledge and technologies, paving the way for developing new treatments, diagnostic techniques, and therapeutic strategies. The equity implications of investing in treatments for rare genetic diseases were not widely considered, and substantially constrains how an investment case can be made given the high upfront costs, especially in low- and middle-income countries. We found limited evidence that these broader factors were considered for RKDs. More generally, economic evaluations are likely to be challenging to conduct in a field often defined by limited clinical data and uncertainty in outcomes.

Combined, these challenges highlight the uncertainties inherent in funding models and health technology assessment processes regarding funding and incentivizing the development of treatments for RKDs. The evidence of this review suggests that though there are special considerations in some systems for treatments for rare diseases, the unique benefits from treatments for RKDs may be systematically underestimated. There might be scope to trial use of alternative funding mechanisms such as public-private partnerships, tax incentives, or building on existing orphan drug price regulations to improve the economic sustainability of treatment development for RKDs.

There were limitations to our review. Although the strengths of the review lies in the broad search strategy and research question, which enabled us to take a comprehensive view of the impact of RKDs on patients and health systems, the heterogeneity of included studies with regard to their varied scope, diverse populations, and health systems restricted our ability to pool their results or conduct a formal meta-analysis of their results and limited the policy implications that could be drawn from the identified literature. Another challenge for this review was the inconsistent reporting of costs. For instance, it was often unclear if costs were incurred by payers or whether they were out-of-pocket. The difficulty of comparing studies across diseases and settings limits our ability to develop an investment case. This aligns with other reviews and supports the call by Marshall and colleagues for a consistent approach to measuring the burden of rare diseases.^11^ A significant proportion of the cost-effectiveness evidence found through this review, however, relates to renal cell carcinomas that as a class are not necessarily rare but include numerous rare inherited syndromes associated with such cancers. Most of the evidence found was produced in high-income nations, most notably the USA, with very limited published evidence on the economic impact of RKDs in low and middle-income countries. Most included studies were funded by pharmaceutical companies, potentially skewing the focus of this evidence away from nonpharmaceutical interventions and overlooking regions of the world where there might be less of a potential market. Although these findings highlight several cost-effective interventions in RKDs, efficient and equitable investment options may have been missed due to remaining gaps in evidence. Although we sought to identify reports in the grey literature, challenges associated with this and the nature of health technology assessment processes around the world mean that some of these could have been missed. Furthermore, the difficulty in separating the economic impact of RKDs on kidneys from other organ systems complicates the interpretation of results, because these diseases often have systemic effects. Finally, we did not conduct any quality appraisal of the included studies.

Future research should address the gaps identified in this review by focusing on low- and middle-income countries, where the economic burden of RKDs may be more pronounced due to limited health care resources. Prospective studies incorporating patient-reported outcomes and broader societal perspectives are needed to better capture the full economic impact of RKDs. In addition, research should explore the cost-effectiveness of nonpharmaceutical interventions, such as lifestyle modifications and early screening programs, which could offer cost-saving opportunities. Efforts to develop comprehensive databases and registries for RKDs would enhance the quality and scope of future economic analyses. Moreover, studies should aim to disentangle the specific economic impacts on the kidneys from impacts on other organ systems to better understand the burden associated with RKDs.

### Conclusions

Our scoping review of economic evaluations, health technology assessments, and cost of illness studies in RKDs has pulled together this evidence base for the first time. Despite evidence of a substantial economic burden facing health systems from RKDs, there are substantial gaps in the economic literature, particularly regarding geographic location and most disease types. Included studies focused on health system costs with limited consideration of broader costs and impacts that could be significant considerations in investment decisions relating to treatment for RKDs.

## Disclosure

DPG reports receiving consulting fees from Novartis, Alexion, Judo Bio, Calliditas, Sanofi, Alnylam, Sofinnova, and Britannia; honoraria payments from Vifor, Sanofi, and Stada. IIU reports honoraria payments from Boehringer Ingelheim, Astra Zeneca and Sanofi. JB reports receiving consulting fees from Alnylam, Argenx, Astellas, BioCryst, Calliditas, Chinook, Dimerix, Galapagos, Novartis, Omeros, Travere Therapeutics, Vera Therapeutics, and Visterra; and grant support from Argenx, Calliditas, Chinook, Galapagos, GlaxoSmithKline, Novartis, Omeros, Travere Therapeutics, and Visterra. DPG is a Trustee and Scientific Advisor for Alport UK, and Chair of UK Kidney Association Rare Diseases Committee. OD is supported by the ITINERARE University Research Priority Program of the University of Zurich and by the European Reference Network for Rare Kidney Diseases (ERKNet) funded by the European Union within the framework of the EU4Health Programme (101085068). BA is supported by a NHMRC Investigator Grant (GNT2010055). ISN provided funding support to The George Institute for Global Health, Australia (AP, AS, BA, SJ, SW, TG) for this research. All the other authors declared no competing interests.

## Supplementary Material

Supplementary File (PDF) **Classification and listing of rare renal diseases. Figure S1.** PRISMA flow chart of study inclusion. **Table S1.** Search strategy for Medline. **Table S2.** Overview of cost-of-illness studies. **Table S3.** Overview of economic evaluation studies. **PRISMA Checklist.**

## Footnotes

Supplementary File (PDF)

**Classification and listing of rare renal diseases.**
**Figure S1.** PRISMA flow chart of study inclusion. **Table S1.** Search strategy for Medline. **Table S2.** Overview of cost-of-illness studies. **Table S3.** Overview of economic evaluation studies.
**PRISMA Checklist.**
