# Supplementary material for: Scoping Review of Economic Analyses of Rare Kidney Diseases
Source: Kidney Int Rep. 2024 Sep 12;9(12):3553–69. doi: 10.1016/j.ekir.2024.09.004 (PMC11652074; doi:10.1016/j.ekir.2024.09.004)
Supplement: Supplementary File (PDF) — Classification and listing of rare renal diseases. Figure S1. PRISMA flow chart of study inclusion. Table S1. Search strategy for Medline. Table S2. Overview of cost-of-illness studies. Table S3. Overview of economic evaluation studies. PRISMA Checklist. [file mmc1.pdf]

Classification and Listing of Rare Renal Diseases

Categories:

1. Genetic cystic renal disease ORPHA:93587
2. Glomerular disease ORPHA:93548
3. Hematological disorder with renal involvement ORPHA:93614
4. Inherited renal cancer-predisposing syndrome ORPHA:319328
5. Nephropathy secondary to a storage or other metabolic disease ORPHA:93593
6. Rare cause of hypertension ORPHA:93618
7. Rare renal tubular disease ORPHA:93603
8. Rare renal tumor ORPHA:93619
9. Renal or urinary tract malformation ORPHA:93545
10. Thrombotic microangiopathy ORPHA:93573
11. Genetic diseases/ syndromes with kidney involvement: Groopman E list

Sub-Classication of Categories

1. Genetic cystic renal disease

- Adult familial nephronophthisis-spastic quadriparesia syndrome ORPHA:2666
- Autosomal dominant polycystic kidney disease ORPHA:730
- Autosomal dominant polycystic kidney disease type 1 with tuberous sclerosis ORPHA:88924
- Autosomal dominant tubulointerstitial kidney disease ORPHA:34149
  - HNF1B-related autosomal dominant tubulointerstitial kidney disease ORPHA:93111
  - MUC1-related autosomal dominant tubulointerstitial kidney disease ORPHA:88949
  - REN-related autosomal dominant tubulointerstitial kidney disease ORPHA:217330
  - UMOD-related autosomal dominant tubulointerstitial kidney disease ORPHA:88950
- Autosomal recessive polycystic kidney disease ORPHA:731
- Bardet-Biedl syndrome ORPHA:110
- Cranioectodermal dysplasia ORPHA:1515
- Ellis Van Creveld syndrome ORPHA:289
- Hepatic fibrosis-renal cysts-intellectual disability syndrome ORPHA:2031
- Joubert syndrome with oculorenal defect ORPHA:2318
- Joubert syndrome with renal defect ORPHA:220497
- Karyomegalic interstitial nephritis ORPHA:401996
- Meckel syndrome ORPHA:564
- Neonatal diabetes-congenital hypothyroidism-congenital glaucoma-hepatic fibrosis-polycystic kidneys syndrome ORPHA:79118
- Nephronophthisis ORPHA:655
  - Infantile nephronophthisis ORPHA:93591
  - Juvenile nephronophthisis ORPHA:93592
  - Late-onset nephronophthisis ORPHA:93589
- Renal-hepatic-pancreatic dysplasia ORPHA:294415
- RHYNS syndrome ORPHA:140976
- Saldino-Mainzer syndrome ORPHA:140969
- Senior-Boichis syndrome ORPHA:84081
- Senior-Loken syndrome ORPHA:3156
- Tuberous sclerosis complex ORPHA:805
- Ventriculomegaly-cystic kidney disease ORPHA:443988

- Von Hippel-Lindau disease ORPHA:892

2. Glomerular Disease

- Collagen-related glomerular basement membrane disease ORPHA:544590
- Alport syndrome ORPHA:63
    - Autosomal dominant Alport syndrome ORPHA:88918
    - Autosomal recessive Alport syndrome ORPHA:88919
    - X-linked Alport syndrome ORPHA:88917
    - X-linked Alport syndrome-diffuse leiomyomatosis ORPHA:1018
    - HANAC syndrome ORPHA:73229
- Disorder with multisystemic involvement and glomerulopathy ORPHA:567562
    - Action myoclonus-renal failure syndrome ORPHA:163696
    - Autosomal dominant intermediate Charcot-Marie-Tooth disease type E ORPHA:93114
    - Congenital membranous nephropathy due to fetomaternal anti-neutral endopeptidase alloimmunization ORPHA:69063
    - Denys-Drash syndrome ORPHA:220
    - Familial steroid-resistant nephrotic syndrome with adrenal insufficiency ORPHA:506334
    - Familial steroid-resistant nephrotic syndrome with sensorineural deafness ORPHA:280406
    - Frasier syndrome ORPHA:347
    - Galloway-Mowat syndrome ORPHA:2065
    - Hypotrichosis-lymphedema-telangiectasia-renal defect syndrome ORPHA:69735
    - Interstitial lung disease-nephrotic syndrome-epidermolysis bullosa syndrome ORPHA:306504
    - MYH9-related disease ORPHA:182050
    - Nail-patella-like renal disease ORPHA:2613
    - Nail-patella syndrome ORPHA:2614
    - Nephrotic syndrome-epidermolysis bullosa-sensorineural deafness syndrome ORPHA:300333
    - Pierson syndrome ORPHA:2670
    - Schimke immuno-osseous dysplasia ORPHA:1830
    - Severe oculo-renal-cerebellar syndrome ORPHA:2715
- Idiopathic non-lupus full-house nephropathy ORPHA:567544
- Nephrotic syndrome without extrarenal manifestations ORPHA:567564
    - Genetic nephrotic syndrome ORPHA:564127
        - Congenital nephrotic syndrome, Finnish type ORPHA:839
        - Genetic steroid-resistant nephrotic syndrome ORPHA:656
    - Idiopathic nephrotic syndrome ORPHA:357502
        - Idiopathic steroid-resistant nephrotic syndrome ORPHA:567548
        - Idiopathic multidrug-resistant nephrotic syndrome ORPHA:567550
        - Idiopathic steroid-resistant nephrotic syndrome with sensitivity to second-line immunosuppressive therapy ORPHA:567552
        - Idiopathic steroid-sensitive nephrotic syndrome ORPHA:69061
        - Idiopathic steroid-sensitive nephrotic syndrome with secondary steroid resistance ORPHA:567546
- Primary membranoproliferative glomerulonephritis ORPHA:54370
    - C3 glomerulopathy ORPHA:329918
        - C3 glomerulonephritis ORPHA:329931
        - Dense deposit disease ORPHA:93571
    - Immunoglobulin-mediated membranoproliferative glomerulonephritis ORPHA:329903
- Primary membranous glomerulonephritis ORPHA:97560
- Systemic disease with glomerulopathy as a major feature ORPHA:567554

- Genetic systemic disease with glomerulopathy as a major feature ORPHA:567556
    - Autoimmune interstitial lung disease-arthritis syndrome ORPHA:444092
    - Familial Mediterranean fever ORPHA:342
    - Fibronectin glomerulopathy ORPHA:84090
    - Hereditary amyloidosis with primary renal involvement ORPHA:85450
    - AApoAI amyloidosis ORPHA:93560
    - AApoAII amyloidosis ORPHA:238269
    - AFib amyloidosis ORPHA:93562
    - ALys amyloidosis ORPHA:93561
    - Hypocomplementemic urticarial vasculitis ORPHA:36412
    - Lipoprotein glomerulopathy ORPHA:329481
    - Muckle-Wells syndrome ORPHA:575
- Non-genetic systemic disease with glomerulopathy as a major feature ORPHA:567558
    - AA amyloidosis ORPHA:85445
    - AApoAIV amyloidosis ORPHA:439232
    - Adult-onset Still disease ORPHA:829
    - AH amyloidosis ORPHA:442582
    - AL amyloidosis ORPHA:85443
    - Behçet disease ORPHA:117
    - Collagen type III glomerulopathy ORPHA:84087
    - Dermatomyositis ORPHA:221
    - IgG4-related kidney disease ORPHA:449395
    - Immunotactoid or fibrillary glomerulopathy ORPHA:91137
    - Immunotactoid glomerulopathy ORPHA:97567
    - Non-amyloid fibrillary glomerulopathy ORPHA:97566
    - Juvenile dermatomyositis ORPHA:93672
    - Juvenile polymyositis ORPHA:93568
    - Mixed connective tissue disease ORPHA:809
    - Non-amyloid monoclonal immunoglobulin deposition disease ORPHA:86861
    - Heavy chain deposition disease ORPHA:93556
    - Light and heavy chain deposition disease ORPHA:93557
    - Light chain deposition disease ORPHA:93558
    - Polymyositis ORPHA:732
    - Reynolds syndrome ORPHA:779
    - Sarcoidosis ORPHA:797
    - Systemic sclerosis ORPHA:90291
    - Diffuse cutaneous systemic sclerosis ORPHA:220393
    - Limited cutaneous systemic sclerosis ORPHA:220402
    - Limited systemic sclerosis ORPHA:220407
    - Systemic vasculitis associated with glomerulopathy ORPHA:567560
    - Anti-glomerular basement membrane disease ORPHA:375
    - Buerger disease ORPHA:36258
    - Cryoglobulinemic vasculitis ORPHA:91138
    - Mixed cryoglobulinemia type II ORPHA:93554
    - Mixed cryoglobulinemia type III ORPHA:93555
    - Eosinophilic granulomatosis with polyangiitis ORPHA:183
    - Giant cell arteritis ORPHA:397
    - Granulomatosis with polyangiitis ORPHA:900
    - Immunoglobulin A vasculitis ORPHA:761
    - Microscopic polyangiitis ORPHA:727
    - Pauci-immune glomerulonephritis ORPHA:93126
    - Pauci-immune glomerulonephritis with ANCA ORPHA:97563

        - Pauci-immune glomerulonephritis without ANCA ORPHA:97564
        - Pediatric systemic lupus erythematosus ORPHA:93552
        - Polyarteritis nodosa ORPHA:767
        - Primary polyarteritis nodosa ORPHA:439737
        - Cutaneous polyarteritis nodosa ORPHA:439729
        - Single-organ polyarteritis nodosa ORPHA:439755
        - Systemic polyarteritis nodosa ORPHA:439762
        - Secondary polyarteritis nodosa ORPHA:439746
        - Relapsing polychondritis ORPHA:728
        - Systemic lupus erythematosus ORPHA:536
        - Takayasu arteritis ORPHA:3287

3. Hematological disorder with renal involvement

- Beta thalassemia
    - Beta thalassemia intermedia
    - Beta thalassemia major
    - Dominant beta thalassemia
- Fanconi anemia
- Sickle cell anemia

4. Inherited renal cancer-predisposing syndrome

- Beckwith Wiedemann syndrome
    - Beckwith Wiedemann syndrome due to 11p15 microdeletion
    - Beckwith Wiedemann syndrome due to 11p15 microduplication
    - Beckwith Wiedemann syndrome due to 11p15 translocation/inversion
    - Beckwith Wiedemann syndrome due to CDKN1C mutation
    - Beckwith Wiedemann syndrome due to imprinting defect of 11p15
    - Beckwith Wiedemann syndrome due to NSD1 mutation
    - Beckwith Wiedemann syndrome due to paternal uniparental disomy of chromosome 11
- Birt-Hogg-Dube syndrome
- Familial papillary thyroid carcinoma with renal papillary neoplasia
- Global developmental delay- lung cysts- overgrowth- Wilms tumor syndrome
- Hereditary clear cell renal cell carcinoma
- Hereditary leiomyomatosis and renal cell cancer
- Hereditary papillary renal cell carcinoma
- Hyperparathyroidism- jaw tumor syndrome
- Inherited cancer- predisposing syndrome due to biallelic BRCA2 mutations
- Perlman syndrome
- Tuberous sclerosis complex
- Von Hippel-Lindau disease
- WAGR syndrome

5. Nephropathy secondary to a storage or metabolic disorder

- Adenine phosphoribosyltransferase deficiency
- Alkaptonuria
- Alpha-1-antitrypsin deficiency
- Amelogenesis imperfecta-nephrocalcinosis syndrome
- Autosomal recessive infantile hypercalcemia
- Congenital disorder of glycosylation with nephropathy as a major feature
    - ALG8-CDG
    - Musculocontractural Ehlers-Danlos syndrome
    - SLC35A1-CDG
- Dent disease

    - Dent disease type 1 (CLCN5-related)
    - Dent disease type 2 (OCRL-related)
- Enamel-renal syndrome
- Fabry disease
- Familial renal glucosuria
- Fanconi-Bickel syndrome
- Galactosemia
    - Classic galactosemia
    - Galactokinase deficiency
    - Galactose epimerase deficiency
        - Erythrocyte galactose epimerase deficiency
        - Generalised galactose epimerase deficiency
    - Galactose mutarotase deficiency
- Gitelman-like kidney tubulopathy due to mitochondrial DNA mutation
- Glycogen storage disease due to glucose-6-phosphatase deficiency
    - Glycogen storage disease due to glucose-6-phosphatase deficiency type Ia
    - Glycogen storage disease due to glucose-6-phosphatase deficiency type Ib
- Glycogen storage disease due to GLUT2 deficiency
- Hartnup disease
- Hereditary fructose intolerance
- Hereditary xanthinuria
    - Xanthinuria type I
    - Xanthinuria type II
- Hypertrophic cardiomyopathy with kidney anomalies due to mitochondrial DNA mutation
- Hypoxanthine-guanine phosphoribosyltransferase deficiency
    - Hypoxanthine-guanine phosphoribosyltransferase partial deficiency
    - Lesch-Nyhan syndrome
- Imerslund-Grasbeck syndrome
- Infantile nephropathic cystinosis
- Juvenile cataract-microcornea-renal glucosuria syndrome
- Juvenile nephropathic cystinosis
- LCAT deficiency
    - Familial LCAT deficiency
    - Fish-eye disease
- Lowe Syndrome
- Lysinuric protein intolerance
- Phosphoribosylpyrophosphate synthetase superactivity
    - Mild phosphoribosylpyrophosphate synthetase superactivity
    - Severe phosphoribosylpyrophosphate synthetase superactivity
- Porphyria
    - Acute hepatic porphyria
        - Acute intermittent porphyria
        - Hereditary coproporphyria
        - Porphyria due to ALA dehydratase deficiency
        - Porphyra variegata
    - Autosomal erythropoietic protoporphyria
    - Chronic hepatic porphyria
        - Hepatoerythropoietic porphyria
        - Porphyria cutanea tarda
    - Congenital erythropoietic porphyria
    - Erythropoietic uroporphyria associated with myeloid malignancy
    - X-linked erythropoetic protoporphyria
- Primary hyperoxaluria
    - Primary hyperoxaluria type 1
    - Primary hyperoxaluria type 2

  - Primary hyperoxaluria type 3
- Sialidosis type 2
  - Congenital sialidosis type 2
  - Juvenile sialidosis type 2
- Tyrosinemia type 1
- Tubulopathy due to mitochondrial oxidative phosphorylation disorder
- Vitamin B12-responsive methylmalonic acidemia
  - Vitamin B12-responsive methylmalonic acidemia type cblA
  - Vitamin B12-responsive methylmalonic acidemia type cblB
  - Vitamin B12-responsive methylmalonic acidemia type cblDv2
- Vitamin B12-unresponsive methylmalonic acidemia
  - Vitamin B12-unresponsive methylmalonic acidemia type mut-
  - Vitamin B12-unresponsive methylmalonic acidemia type mut0
- Wilson disease
- Zellweger syndrome

6. Rare causes of Hypertension
- Congenital renal artery stenosis
- Apparent mineralocorticoid excess
- Autosomal dominant progressive nephropathy with hypertension
- Brachydactyly-arterial hypertension syndrome
- William syndrome
- Pseudoxanthoma elasticum
- Liddle syndrome
- Familial hyperthyroidism due to mutation in TSH receptor
- Hypertension due to gain of function mutation in the mineralocorticoid receptor
- Liddle syndrome
- Familial Hyperaldosteronism (types 1, 2, 3)
- Neurofibromatosis type 1
  - 17q11 microdeletion syndrome ORPHA:97685
  - Neurofibromatosis type 1 due to NF1 mutation or intragenic deletion
- Pseudohypoaldosteronism type 2 (2A,2B, 2C, 2D, 2E)
- Pseudoxanthoma elasticum
- Williams Syndrome

7. Rare renal tubular disease

- Alstrom syndrome
- Acquired monoclonal Ig light chain associated Fanconi syndrome
- Atypical Fanconi syndrome-neonatal hyperinsulinism syndrome
- Autosomal dominant primary hypomagnesemia with hypocalciuria
- Cataract nephropathy encephalopathy syndrome
- Bartter syndrome type 1,2,3,4,5
- Cystinuria type A, B
- Dominant hypophosphatemia with nephrolithiasis or osteoporosis
- EAST syndrome
- EGF related primary hypomagnesemia with intellectual disability
- Gitelman like kidney tubulopathy due to mitochondrial DNA mutation
- Gitelman syndrome
- Hereditary renal hypouricemia
- Hypertrophic cardiomyopathy with kidney anomalies due to mitochondrial DNA mutation
- Hyperuricemia pulmonary hypertension- renal failure- alkalosis syndrome
- Hypohidrosis- electrolyte imbalance-lacrimal gland dysfunction- icthyosis- xerostoia syndrome
- Hypophosphatemic rickets

    - Autosomal dominant hypophosphatemic rickets
    - Autosomal recessive hypophosphatemic rickets
    - Dent disease (type 1,2)
    - Dominant hypophosphatemia with nephrolithiasis or osteoporosis
    - Hereditary hypophosphatemic rickets with hypercalciuria
    - X-linked hypophosphatemia
- Hypotonia- cystinuria type 1 syndrome
    - 2p21 microdeletion syndrome
    - Hypotonia-cystinuria syndrome
- Idiopathic hypercalciuria
- Isolated autosomal dominant hypomagnesemia, Glaudemans type
- Jeune syndrome
- Kidney tubulopathy-dilated cardiomyopathy syndrome
- Mitochondrial DNA depletion syndrome, hepatocerebrorenal form
- Nephrogenic Diabetes insipidus
- Nephrogenic diabetes insipidus-intracranial calcification- short stature-facial dysmorphism syndrome
- Nephrogenic syndrome of inappropriate diuresis
- Oculocerebrorenal syndrome of Lowe
- Oncogenic osteomalacia
- Primary Fanconi renotubular syndrome
- Primary hypomagnesemia-generalized seizure -intellectual disability-obesity syndrome
- Primary Hypomagnesemia- refractory seizure-intellectual disability syndrome
- Primary hypomagnesemia with hypercalciuria and nephrocalcinosis
    - With and without severe ocular involvement
- Primary hypomagnesemia with secondary hypocalcemia
- Renal Tubular Acidosis
    - Proximal renal tubular acidosis
    - Distal renal tubular acidosis
    - Osteopetrosis with renal tubular acidosis
    - Central nervous system calcification-deafness-tubular acidosis- anemia syndrome
- Pseudohypoaldosteronism type 1,2
- Pseudohypoparathyroidism
    - With and Without Albright hereditary osteodystrophy
- Psychomotor regression-oculomotor apraxia-movement disorder-nephropathy syndrome
- Tubulointerstitial nephritis and uveitis syndrome

8. Rare Renal Tumors

- Benign metanephric tumor
- Clear cell sarcoma of kidney
- Congenital mesoblastic nephroma
- Cystic hamartoma of lung and kidney
- Multiloculated renal cyst
- Nephroblastoma
- Renal cell carcinoma
- Acquired cystic disease -associated renal cell carcinoma
- Chromophobe renal cell carcinoma
- Collecting duct carcinoma
- MiT family translocation renal cell carcinoma
- Mucinous tubular and spindle cell renal carcinoma
- Papillary renal cell carcinoma
- Renal medullary carcinoma
- Tubulocystic renal cell carcinoma

- Clear cell renal carcinoma
- Clear cell papillary renal cell carcinoma
- Multilocular cystic renal neoplasm of low malignant potential

9. Renal or urinary tract malformation

Non-syndromic renal or urinary tract malformation:

- Congenital hydronephrosis
  - Congenital bilateral megacalycosis
  - Unilateral congenital megacalycosis
- Ureteropelvic junction (UPJ) obstruction bilateral or in solitary kidney
- Congenital primary megaureter, nonrefluxing and unobstructed form
- Congenital primary megaureter, obstructed form
- Congenital primary megaureter, refluxing and obstructed form
- Congenital primary megaureter, refluxing form
- Primary megaureter, adult-onset form
- Patent urachus
- Urachal cyst
- Urachal diverticulum
- Urachal sinus
- Duplication of urethra
- Exstrophy-epispadias complex
- Bladder exstrophy
- Cloacal exstrophy
- Isolated epispadias
- Familial vesicoureteral reflux
- Vesicoureteral reflux, high grade
- Anterior urethral valve
- Atresia of urethra
- Posterior urethral valve
- Prune belly syndrome
- Congenital hydronephrosis
- Medullary sponge kidney
- Megacystis-megaureter syndrome
- Bilateral multicystic dysplastic kidney
- Unilateral multicystic dysplastic kidney
- Oligomeganephronia
- Bilateral Renal agenesis
- Unilateral Renal agenesis
- Renal dysplasia, bilateral
- Renal dysplasia, unilateral
- Renal hypoplasia, bilateral
- Renal hypoplasia, unilateral
- Drug-related renal tubular dysgenesis
- Renal tubular dysgenesis due to twin-twin transfusion
- Renal tubular dysgenesis of genetic origin
- Neurogenic bladder, congenital or acquired form

Syndromic renal or urinary tract malformation:

- 22q11.2 deletion syndrome (Di George syndrome)
- 8q24.3 microdeletion syndrome
- Acrorenal syndrome
- Acroosteolysis dominant type

- Alagille syndrome due to 20p12 microdeletion
- Alagille syndrome due to a JAG1 point mutation
- Alagille syndrome due to a NOTCH2 point mutation
- Aniridia-renal agenesis-psychomotor retardation syndrome
- AREDYLD (acral-renal-ectodermal-dysplasia-lipoatrophic-diabetes) syndrome
- Arthrogryposis-renal dysfunction-cholestasis syndrome
- Atherosclerosis-deafness-diabetes-epilepsy-nephropathy syndrome
- Axial mesodermal dysplasia spectrum
- Bardet-Biedl syndrome (renal/urinary tract malformation) – types 1-12, 17-19
- Beckwith-Wiedemann syndrome due to 11p15 microdeletion
- Beckwith-Wiedemann syndrome due to 11p15 microduplication
- Beckwith-Wiedemann syndrome due to 11p15 translocation/inversion
- Beckwith-Wiedemann syndrome due to CDKN1C mutation
- Beckwith-Wiedemann syndrome due to imprinting defect of 11p15
- Beckwith-Wiedemann syndrome due to NSD1 mutation
- Beckwith-Wiedemann syndrome due to paternal uniparental disomy of chromosome 11
- BNAR (Bifid nose, anorectal and renal anomalies) syndrome
- BOR (branchio-oto-renal) syndrome
- Cat-eye syndrome
- Caudal regression syndrome
- CHARGE syndrome
- Congenital vertebral-cardiac-renal anomalies syndrome
- Cornelia de Lange syndrome
- Cystic hamartoma of lung and kidney
- Double uterus-hemivagina-renal agenesis syndrome
- Dyschondrosteosis-nephritis syndrome
- EEC (Ectrodactyly-ectodermal dysplasia-cleft lip/palate) syndrome
- Ellis Van Creveld syndrome
- Faciocardiorenal syndrome
- Fibulo-ulnar hypoplasia-renal anomalies syndrome
- Fraser syndrome
- Hajdu-Cheney syndrome
- HNF1B-related autosomal dominant tubulointerstitial kidney disease
- Holoprosencephaly-radial heart renal anomalies syndrome
- Hydrocephalus-blue sclerae-nephropathy syndrome
- Hypoparathyroidism-sensorineural deafness-renal disease syndrome
- Infundibulopelvic stenosis-multicystic kidney syndrome
- Ichthyosis-intellectual disability-dwarfism-renal impairment syndrome
- Jeune syndrome (renal/urinary tract malformation)
- Kabuki Syndrome
- Kallmann syndrome
- Lethal fetal brain malformation-duodenal atresia-bilateral renal hypoplasia syndrome
- Lethal fetal cerebrorenogenitourinary agenesis/hypoplasia syndrome
- Mayer-Rokitansky-Küster-Hauser syndrome type 1
- Mayer-Rokitansky-Küster-Hauser syndrome type 2
- Meckel syndrome
- Megacystis-microcolon-intestinal hypoperistalsis syndrome
- Menke-Hennekam syndrome
- Multicentric carpo-tarsal osteolysis with nephropathy
- Multinucleated neurons-anhydramnios-renal dysplasia-cerebellar hypoplasia-hydranencephaly syndrome
- Nephrosis-deafness-urinary tract-digital malformations syndrome

- Neurofaciodigitorenal syndrome
- Noonan syndrome
- NPHP3-related Meckel-like syndrome
- Ochoa syndrome
- Orofaciodigital syndrome type 1
- Pallister-Hall syndrome
- Renal cysts and diabetes syndrome
- Renal caliceal diverticuli-deafness syndrome
- Renal coloboma syndrome
- Renal nutcracker syndrome
- Rubinstein-Taybi syndrome due to 16p13.3 microdeletion
- Rubinstein-Taybi syndrome due to CREBBP mutations
- Rubinstein-Taybi syndrome due to EP300 haploinsufficiency
- Schinzel-Giedion syndrome
- SERKAL syndrome
- SETD2-related microcephaly-severe intellectual disability-multiple congenital anomalies syndrome
- Simpson-Golabi-Behmel syndrome
- Smith-Lemli-Opitz syndrome
- Spastic paraplegia-nephritis-deafness syndrome
- Tall stature-intellectual disability-renal anomalies syndrome
- Thomas syndrome
- Thymic-renal-anal-lung dysplasia
- Thyrocerebrorenal syndrome
- Townes-Brocks syndrome
- Trisomy 13
- Trisomy 18
- Turner syndrome
- Monosomy X
- Mosaic monosomy X
- Turner syndrome due to structural X chromosome anomalies
- Ulbright-Hodes syndrome
- VACTERL/VATER association
- WAGR syndrome

10. Thrombotic Microangiopathy

- De novo thrombotic microangiopathy after kidney transplantation
- Atypical hemolytic uremic syndrome with anti-factor H antibodies
- Atypical hemolytic uremic syndrome with complement gene abnormality
    - Complement factor H, I, 3, 4A deficiency
- Atypical hemolytic uremic syndrome not otherwise specified
- Hemolytic uremic syndrome with DGKE deficiency
- Infection-related hemolytic uremic syndrome
- Shiga toxin-associated hemolytic uremic syndrome
- Streptococcus pneumoniae-associated hemolytic uremic syndrome
- Methylmalonic acidemia with homocystinuria, type cblC
- Pediatric systemic lupus erythematosus
- Systemic lupus erythematosus associated TMA
- Thrombotic thrombocytopenic purpura
- Congenital thrombotic thrombocytopenic purpura
- Immune-mediated thrombotic thrombocytopenic purpura
- Acquired thrombotic thrombocytopenic purpura

11. Genetic diseases/ syndromes with kidney involvement: Groopman E list

- Coenzyme q10 deficiency
- Coloboma, ocular` with or without hearing impairment, cleft lip palate, and or mental retardation
- Coffin Siris syndrome
- Cockayne syndrome
- Chondroplasia Punctata syndrome
- Cardiofaciocutaneous syndrome
- Burn-McKeown syndrome
- Blackfan-Diamond anemia
- Adrenal hypoplasia, congenital, with hypogonadotropic hypogonadism
- Alkaptonuria
- Adrenal hypoplasia, congenital, with hypogonadotropic hypogonadism
- Alagille syndrome type 1
- Alkaptonuria
- Acro-renal-ocular syndrome, Duane-Radial Ray Syndrome IVIC Syndrome
- 5-oxoprolinase deficiency
- Arthrogryposis, renal dysfunction, and cholestasis 2` arcs2
- Atypical Werner syndrome Laminopathy type Decaudain-Vigouroux LMNA-related cardiocutaneous progeria syndrome, Restrictive Dermopathy, Lethal
- Autoimmune Disease, multisystem, infantileJonset, 2
- Autoimmune interstitial lung, joint, and kidney disease
- Autoimmune Lymhoproliferative syndrome type 2A
- Autosomal dominant periodic fever syndrome
- Autosomal recessive polycystic kidney disease, Polycystic and hepatic disease
- Autosomal recessive spondylocostal dysostosis
- Hypotrichosis-lymphedema-telangiectasia-renal defect syndrome
- Hypouricemia, renal, 1, rhuc1
- Hypouricemia, renal, 2
- IFAP Syndrome with or without Bresheck Syndrome
- Immunodeficiency 23
- Immunodeficiency-centromeric instability, facial anomalies syndrome 1, icf1
- Immunodeficiency, common variable, 3,6
- Hypomagnesemia associated with myokymia
- Hypomagnesemia type 6 Hypomagnesemia, seizures, and mental retardation
- Hypoparathyroidism-retardation-dysmorphism syndrome` Kenny-Caffey syndrome, type 1
- Hypoparathyroidism, deafness, renal disease syndrome
- Hereditary hemorrhagic telangiectasia
- Hereditary orotic aciduria
- Hermansky-Pudlak syndrome 1
- HERNS syndrome` Vasculopathy, retinal, with cerebral leukodystrophy
- Hydrolethalus syndrome
- Hydroxykynureninuria
- Hyperaldosteronism,familial,type 3
- Hypercalcemia infantile, 1
- Hyperglycinuria
- GM1-gangliosidosis, type 1, type 2
- Hand-foot-uterus syndrome
- Hartnup Disorder Hyperglycinuria
- Hemorrhagic destruction of the brain, subependymal calcification, and cataracts

- Hennekam Lymphangiectasia-Lymphedema Syndrome
- Hereditary angiopathy with nephropathy, aneurysms and Muscle cramps (HANAC)
- Glomerulopathy with fibronectin deposits 2
- Glomerulosclerosis, focal segmental, 6
- Glutaric acidemia 2b
- Glutaric acidemia 2c
- Glutaric aciduria 2a
- Glutaric aciduria 3
- Glutaricaciduria, type I
- Galloway-Mowat syndrome
- Gaucher disease
- Genitopatellar syndrome
- Gillessen-Kaesbach-Nishimura sundrome (GIKANIS), Congenital disorder of glycosylation, type 2
- Focal Segmental Glomerulosclerosis 5 Charcot-Marie-Tooth disease E
- Fanconi anemia, complementation group a
- Fanconi anemia, complementation group b
- Fanconi anemia, complementation group c
- Fanconi anemia, Complementation group d2
- Fanconi anemia, complementation group e
- Fanconi anemia, complementation group I
- Fanconi anemia, complementation group L
- Fanconi Anemia, Complementation Group N
- Fanconi anemia, complementation group O
- Fanconi anemia, complementation group P
- Fanconi anemia, Xeroderma pigmentosum, type f, Cockayne syndrome
- Fanconi renotubular syndrome 2 hypercalcemia, infantile, 2 nephrolithiasis,osteoporosis, hypophosphatemic, 1
- Fanconi renotubular syndrome 3
- Fanconi-Bickel syndrome
- Fechtner syndrome` Epstein Syndrome
- Feingold syndrome
- Feingold syndrome 2` Brachydactyly with short stature and microcephaly
- Floating-Harbor syndrome
- Focal dermal hypoplasia
- Diaphanospondylodysostosis
- Diarrhea 3, secretory sodium, congenital, syndromic
- Dicarboylic aminoaciduria
- Donnai-barrow syndrome
- DOOR syndrome
- Dyskeratosis congenita
- Dyskeratosis congenita
- Dyskeratosis Congenita, X-linked
- EEC syndrome, Ectrodactyly, ectodermal dysplasia, and cleft lip,palate syndrome 3
- Ehlers-Danlos syndrome with progressive kyphoscoliosis, myopathy, and hearing loss
- Ehlers-Danlos syndrome, classic type
- Ehlers-Danlos syndrome, musculocontractural type 1
- Ehlers-Danlos syndrome, type 6
- Ellis Van Creveld syndrome
- Ellis Van Creveld syndrome
- Encephalocraniocutaneous Lipomatosis, Kallmann syndrome 2, kal2
- Endocrine-cerebroosteodysplasia

- Epidermolysis bullosa, junctional, Herlitz type` Epidermolysis bullosa, junctional, non-Herlitz type
- Epidermolysis bullosa, junctional, with pyloric atresia
- Epilepsy, Progressive Myoclonic, 4 with or without Renal Failure
- Even-plus syndrome
- Fabry Disease
- Familial dysautonomia, Hereditary sensory and autonomic neuropathy type 3, hsan3
- Familial Mediterranean fever
- Developmental delay with short stature, dysmorphic features, and sparse hair
- Diabetes mellitus, insulin-dependent, 20` MODY, type 3` Renal cell carcinoma
- Costello syndrome
- Cowden syndrome
- Cranioectodermal dysplasia type 1
- Cranioectodermal dysplasia type 3
- Craniofacial dysmorphism, skeletal anomalies, and mental retardation syndrome
- Currarino syndrome
- Cushing syndrome due to macronodular adrenal hyperplasia
- Cutis laxa type 1, 1A, 1C
- Cystinosis, nephropathic cystinosis, late-onset Juvenile or adolescent nephropathic
- Cystinuria
- D-bifunctional protein deficiency
- Infantile myofibromatosis, Cerebral arteriopathy with subcortical infarcts and leukoencephalopathy 1, Lateral meningocele syndrome
- Interstitial lung disease, nephrotic syndrome, and epidermolysis bullosa, congenital
- Interstitial nephritis, karyomegalic
- Johanson-Blizzard syndrome
- Joubert syndrome 16
- Joubert syndrome 18, Orofaciodigital syndrome 4
- Joubert syndrome 2, Meckel syndrome
- Joubert syndrome 25
- Joubert Syndrome 3
- Joubert syndrome type 10, Orofaciodigital syndrome I, Golabi-Behmel syndrome, type 2
- Joubert syndrome type 14
- Joubert syndrome type 20, meckel-gruber syndrome type 11
- Joubert syndrome with hepatic defect, Joubert syndrome with oculorenal defect, Meckel Syndrome 6, Jouber syndrome 9, COACH syndrome
- Joubert syndrome with hepatic defectJoubert Syndrome 1
- Joubert syndrome with oculorenal defect, BardetJbiedl syndrome 14, Joubert Syndrome 5, Meckel syndrome 4, SeniorJLoken Syndrome 6
- Joubert's syndrome type 15
- Joubert's syndrome type 22
- Junctional epidermolysis bullosa-pyloric atresia syndrome, Epidermolysis bullosa, junctional, with pyloric stenosis
- Kabuki syndrome
- Kabuki syndrome
- Kabuki syndrome
- Kabuki syndrome 1
- Kallmann syndrome 3, kal3 (hypogonadotropic hypogonadism 3 with or without anosmia)
- Kelley-Seegmiller Syndrome Lesch-Nyhan Syndrome
- Knobloch syndrome
- Koolen-De Vries Syndrome
- Lacrimoauriculodentodigital syndrome

- Lathosterolosis
- Laurence-Moon syndrome
- Lethal congenital contracture syndrome 2
- Lethal neonatal carnitine palmitoyltransferase 2 deficiency
- Liddle syndrome
- Linear skin defects with multiple congenital anomalies 2
- Lipodystrophy congenital generalized, type 1
- Lipodystrophy congenital generalized, type 2
- Lipoid adrenal hyperplasia
- Loeys-Dietz syndrome 3
- Lymphedema-distichiasis syndrome with renal disease and diabetes mellitus
- Lymphedema, hereditary, IA
- Lysinuric protein intolerance
- Macrocephaly, alopecia, cutis laxa, and scoliosis
- Mandibulfacial dysostosis with alopecia
- Marden-Walker Syndrome
- McArdle Disease
- Meckel syndrome 1, Bardet-Biedl syndrome 13
- Meckel syndrome 12
- Meckel syndrome 5, Joubert syndrome 7, COACH syndrome
- Meckel syndrome type 10
- Meckel syndrome, Joubert syndrome 21
- Meckel syndrome` Meckel-Gruber syndrome type 9
- Meckel-gruber syndrome type 8, -oubert syndrome 24
- Medullary cystic kidney disease 1
- Medullary cystic kidney disease 2 hyperuricemic nephropathy glomerulocystic kidney disease
- Megacystis-microcolon-intestinal hypoperistalsis syndrome, Visceral myopathy
- Megalencephaly-polymicogyria-polydactyly-hyodrcephalus syndrome 1
- Megaloblastic anemia 1-finnish type
- Megaloblasticanemia1-norwegian_type
- Mental retardation, autosomal dominant 42
- Mental retardation, autosomal dominant, 27
- Mental retardation, x-linked 90
- Mental retardation, X-linked 99, syndromic, female-restricted
- Mental retardation, XJlinked 98
- Metachromatic leukodystrophy due to SAP-b deficiency
- Metaphyseal chondrodysplasia, Murk Jansen type
- Methylmalonic aciduria and homocystinuria, cbl- type
- Methylmalonic aciduria and homocystinuria, cblc type
- Methylmalonic aciduria and homocystinuria, cblF type
- Mevalonic aciduria
- Micro syndrome
- Microcephalic osteodysplastic primordial dwarfism, type I
- Microcephaly, short stature, and impaired glucose metabolism 2
- Microphthalmia with limb anomalies
- Microphthalmia, Lenz type, Microphthalmia, syndromic 1
- Microphthalmia, Lenz type` Microphthalmia, syndromic 2
- Microphthalmia, syndromic 6
- Microphthalmia, syndromic 9, mcops9
- Microsephaly, short stature, and polymicogyria with or without seizures
- Mitochondrial Complex 4 deficiency

- Mitochondrial complex 3 deficiency, nuclear type 1
- Mitochondrial complex 3 deficiency, nuclear type 7
- Mitochondrial Complex 4 deficiency
- Mitochondrial DNA depletion syndrome 13
- Mitochondrial DNA depletion syndrome 8A
- Mitochondrial dna depletion syndrome, encephalomyopathic form, with methylmalonic aciduria
- Mosaic variegated aneuploidy syndrome 1
- Muckle-wells syndrome Familial cold-induced inflammatory syndrome 1
- Muir-Torre syndrome colorectal cancer, hereditary, nonpolyposis, type 1
- Multicentric carpo-tarsal osteolysis with or without nephropathy
- Multiple congenital anomalies-hypotonia-seizures syndrome 1,2,3
- Multiple endocrine neoplasia, type 4
- Multisystemic smooth muscle dysfunction syndrome
- Muscular dystrophy-dystroglycanopathy (congenital with brain and eye anomalies), type A, 13
- Muscular dystrophy-dystroglycanopathy, type A, 1
- Myoglobinuria, acute recurrent
- Myotubular myopathy
- Nager syndrome, Acrofacial dysostosis 1, Nager type
- Nail-patella syndrome
- Neonatal severe primary hyperparathyroidism` Hypocalcemia with Bartter syndrome
- Nephrogenic syndrome of inappropriate diuresis Diabetes insipidus, nephrogenic
- Nephrolithiasis,osteoporosis, hypophosphatemic, 2
- Nephronophthisis 1 - 19
- Nephronophthisis 4 Senior-Loken syndrome 4
- NephronophthisisJ14 Joubert syndrome 16
- Meckel Syndrome 3, Joubert syndrome 6, COACH syndrome
- Nephronophthisis, Joubert syndrome 8
- Nephropathy due to CFHR5 deficiency
- Nephropathy with pretibial epidermolysis bullosa and deafness
- Neu-Laxova syndrome 1
- Neurodevelopmental disorder with or without anomalies of the brain, eye, or heart
- Neurofibromatosis, type I
- Nijmegen breakage syndrome
- Noonan syndrome with multiple lentigines, Cardiofaciocutaneous syndrome
- Noonan syndrome with multiple lentigines Leopard syndrome 1
- Norum disease
- Obesity, adrenal insufficiency, and red hair due to POMC deficiency
- Occipital horn syndrome
- Ochoa syndrome, Urofacial Syndrome 1,2
- Opitz GBBB syndrome, type 2
- Opsismodysplasia
- Orofacial cleft 15
- Orofaciodigital syndrome 5,6
- Pendred syndrome
- Perlman's syndrome (nephroblastomatosis, gigantism)
- Peters-plus syndrome
- Peutz--eghers syndrome
- Pfeiffer syndrome type 3, Antley-Bixler syndrome, Apert syndrome, LADD syndrome
- Pheochromocytoma Cowden syndrome 3
- Phosphoglycerate kinase 1 deficiency

- Phosphoribosylpyrophosphate Synthetase Syperactivity
- Plasminogen deficiency, Type I
- Polycystic kidney disease 1
- Polycystic liver disease 3 with or without kidney cysts
- Polyhydramnios, megalencephaly, and symptomatic epilepsy
- Popliteal pterygium syndrome, Bartsocas-Papas type
- Proud syndrome
- Prune belly syndrome, Eagle-barrett syndrome
- Rabson-Mendenhall syndrome
- Raine syndrome
- Renal agenesis, bilateral
- Renal coloboma syndrome, FSGS 7, Papillorenal Syndrome, PAPRS
- Renal Cysts and Diabetes Syndrome
- Renal cysts and diabetes syndrome` Hyperinsulinism due to HNF4A deficiency` Fanconi renotubular syndrome 4, with maturity-onset diabetes of the young
- Renal glucosuria
- Renpenning Syndrome 1
- Restrictive Dermopathy, Lethal
- Roberts syndrome
- Robinow syndrome
- Robinow syndrome, autosomal dominant 1
- Rubinstein Taybi syndrome type 1
- Scalp-Ear-Nipple Syndrome
- Schimke immuno-osseous dysplasiaSchimke's immunoosseous dystrophy
- Schinzel-Giedion Midface Retraction Syndrome
- Schwartz--ampel syndrome
- Seckel Syndrome 2
- Seckel syndrome 8
- Seckel syndrome 9
- Seizures, sensorineural deafness, ataxia, mental retardation, and electrolyte imbalance (sesame syndrome)
- Senior loken syndrome type 7, Bardet-Biedl Syndrome 16
- Senior-loken syndrome 5
- Senior-Loken syndrome 9
- Senior-Loken syndrome, Nephronophthisis 2, infantile
- Senior-Loken syndrome, Nephronophthisis 3 (adolescent), Meckel syndrome 7, Renal-hepatic-pancreatic dysplasia
- Senior-Loken syndrome, Nephronopthisis 15
- Senior-Loken syndrome, Short-rib thoracic dysplasia 5, Nephronophthisis type 13, Cranioectodermal dysplasia
- SERKAL syndrome Mullerian aplasia and hyperandrogenism
- Short rib-polydactyly syndrome (-eune's syndrome) type 9
- Short rib-polydactyly syndrome, Verma-Naumoff type
- Short rib-polydactyly syndrome, Verma-Naumoff type, Short rib-polydactyly syndrome (-eune's syndrome) type 3
- Short rib-polydactyly syndrome, Verma-Naumoff type, Short rib-polydactyly syndrome (-eune's syndrome) type 7,Cranioectodermal dysplasia type 2
- Short rib-polydactyly syndrome, Verma-Naumoff type, Short rib-polydactyly syndrome (-eune's syndrome) type 8
- Short rib-polydactyly syndrome, Verma-Naumoff typeShort-rib thoracic dysplasia 11 with or without polydactyly
- Short ribJpolydactyly syndrome (Jeune's syndrome) type 6

- Short stature, microcephaly, and endocrine dysfunction
- Short-rib thoracic dysplasia 13 with or without polydactyly
- Shwachman-Diamond syndrome
- Sickle Cell Anemia beta-thalassemia, dominant inclusion body type
- Sideroblastic anemia with B-cell immunodeficiency, periodic fevers, and developmental delay
- Simpson golabi behmel syndrome type 1
- Single median maxillary central incisor holoprosencephaly 3
- Smith-Lemli-Opitz Syndrome
- Smith-Magenis syndrome Yuan-Harel-Lupski syndrome
- Sneddon syndrome Polyarteritis nodosa, childhood-onset
- Sotos syndrome
- Sotos syndrome 1` Beckwith-Wiedemann syndrome, BWS
- Spinocerebellar ataxia, autosomal recessive 16
- Spondylocarpotarsal synostosis syndrome
- Spondylocular syndrome
- Spondyloenchondrodysplasia with immune dysregulation
- STAR (toe Syndactyly, Telecanthus, and Anogenital and Renal Malformations)
- Stromme syndrome
- Sucrase-isomaltase deficiency, congenital
- Symmetric circumferential skin creases congenital, 2
- TARP syndrome
- Tetraamelia-multiple malformations syndrome
- Thanatophoric dysplasia type 1, Thanatophoric dysplasia type 2, Lacrimoauriculodentodigital syndrome
- Thrombocytopenia-absent radius syndrome
- Thrombotic thrombocytopenic purpura, familial
- TMEM70-related mitochondrial encephalo-cardio-myopathy
- Townes-Brocks Branchiootorenal-like Syndrome
- Trichohepatonenteric syndrome 1
- Tuberous Sclerosis-1,2
- Tumoral calcinosis, hyperphosphatemic, familial, hftc
- tyrosinemia, type 1
- Ulna and fibula, absense of, with severe limb deficiency
- Vacterl association with hydrocephalus
- VACTERL association, XJlinked Heterotaxy, visceral, 1
- Van Maldergem syndrome 1
- Van Maldergem Syndrome 2 Hennekam lymphangiectasia-lymphedema syndrome 2
- Verheij syndrome
- Vesicoureteral Reflux 8 Ehlers-Danlos syndrome due to tenascin-X deficiency
- Vici syndrome
- Vitamin B12-unresponsive methylmalonic acidemia type mut0
- Von Hippel-Lindau Syndrome
- Webb-Dattani syndrome
- Wilson Disease
- Wiskott-Aldrich syndrome
- Wolcott-Rallison Syndrome
- Wolfram syndrome 2
- Wolfram syndrome type 1

Table S1 – Search strategy for Medline

	Search terms	No. of hits
#1	(Costs and Cost Analysis[MeSH Terms] OR "cost-minimisation"[title/abstract] OR "cost-minimization"[title/abstract] OR "health technology assessment"[Title/Abstract] OR "cost-effectiveness"[Title/Abstract] OR "cost-utility"[Title/Abstract] OR "cost analysis"[title/abstract] OR "budget impact"[Title/Abstract] OR "cost-benefit"[Title/Abstract])	311,992
#2	(((((((("Shwachman-Diamond syndrome"[Title/Abstract] OR "Sickle Cell Anemia beta-thalassemia"[Title/Abstract] OR "Simpson golabi behmel syndrome type 1"[Title/Abstract] OR "Smith-Lemli-Opitz Syndrome"[Title/Abstract] OR "Sotos syndrome"[Title/Abstract] OR "Spondylocarpotarsal synostosis syndrome"[Title/Abstract] OR "Spondyloenchondrodysplasia with immune dysregulation"[Title/Abstract] OR "toe Syndactyly"[Title/Abstract] OR "Stromme syndrome"[Title/Abstract] OR "TARP syndrome"[Title/Abstract] OR "Thrombocytopenia-absent radius syndrome"[Title/Abstract] OR "Thrombotic thrombocytopenic purpura"[Title/Abstract] OR "Tuberous Sclerosis"[Title/Abstract] OR "tyrosinemia type 1"[Title/Abstract] OR "Vacterl association with hydrocephalus"[Title/Abstract] OR "Verheij syndrome"[Title/Abstract] OR "Vici syndrome"[Title/Abstract] OR "Von Hippel-Lindau Syndrome"[Title/Abstract] OR "Wilson Disease"[Title/Abstract] OR "Wiskott-Aldrich syndrome"[Title/Abstract] OR "Wolcott-Rallison Syndrome"[Title/Abstract] OR "Wolfram syndrome 2"[Title/Abstract] OR "Wolfram syndrome type 1"[Title/Abstract]) OR ("Microphthalmia with limb anomalies"[Title/Abstract] OR "Microphthalmia syndromic 9"[Title/Abstract] OR "Mitochondrial DNA depletion syndrome 13"[Title/Abstract] OR "Mitochondrial DNA depletion syndrome 8A"[Title/Abstract] OR "Mitochondrial dna depletion syndrome encephalomyopathic form with methylmalonic aciduria"[Title/Abstract] OR "Multiple endocrine neoplasia type 4"[Title/Abstract] OR "Multisystemic smooth muscle dysfunction syndrome"[Title/Abstract] OR "Muscular dystrophy-dystroglycanopathy"[Title/Abstract] OR "Myotubular myopathy"[Title/Abstract] OR "Nail-patella syndrome"[Title/Abstract] OR "Neurofibromatosis type I"[Title/Abstract] OR "Nijmegen breakage syndrome"[Title/Abstract] OR "Norum disease"[Title/Abstract] OR "Occipital horn syndrome"[Title/Abstract] OR "Opsismodysplasia"[Title/Abstract] OR "Pendred syndrome"[Title/Abstract] OR "Peters-plus syndrome"[Title/Abstract] OR "Phosphoglycerate kinase 1 deficiency"[Title/Abstract] OR "Plasminogen deficiency Type I"[Title/Abstract] OR "Polycystic kidney disease 1"[Title/Abstract] OR "Polyhydramnios"[Title/Abstract] OR "Popliteal pterygium syndrome"[Title/Abstract] OR "Proud syndrome"[Title/Abstract] OR "Rabson-Mendenhall syndrome"[Title/Abstract] OR "Raine syndrome"[Title/Abstract] OR "Renal agenesis bilateral"[Title/Abstract] OR "Renal glucosuria"[Title/Abstract] OR "Roberts syndrome"[Title/Abstract] OR "Robinow syndrome"[Title/Abstract] OR "Scalp-Ear-Nipple Syndrome"[Title/Abstract] OR "sesame syndrome"[Title/Abstract])) OR ("EEC syndrome"[Title/Abstract] OR "Ectrodactyly ectodermal dysplasia and cleft lip palate syndrome 3"[Title/Abstract] OR "Ehlers-Danlos syndrome classic type"[Title/Abstract] OR "Ehlers-Danlos syndrome	332,861

	musculocontractural type 1"[Title/Abstract] OR "Ehlers-Danlos syndrome type 6"[Title/Abstract] OR "Ellis Van Creveld syndrome"[Title/Abstract] OR "Endocrine-cerebroosteodysplasia"[Title/Abstract] OR "Epidermolysis bullosa junctional"[Title/Abstract] OR "Even-plus syndrome"[Title/Abstract] OR "Fabry Disease"[Title/Abstract] OR "Costello syndrome"[Title/Abstract] OR "Cowden syndrome"[Title/Abstract] OR "Currarino syndrome"[Title/Abstract] OR "Cystinuria"[Title/Abstract] OR "D-bifunctional protein deficiency"[Title/Abstract] OR "Infantile myofibromatosis"[Title/Abstract] OR "Lateral meningocele syndrome"[Title/Abstract] OR "Johanson-Blizzard syndrome"[Title/Abstract] OR "Orofaciodigital syndrome 4"[Title/Abstract] OR "Joubert syndrome 2"[Title/Abstract] OR "Meckel syndrome"[Title/Abstract] OR "Joubert Syndrome 3"[Title/Abstract] OR "Joubert syndrome type 10"[Title/Abstract] OR "Kabuki syndrome"[Title/Abstract] OR "Kabuki syndrome 1"[Title/Abstract] OR "Kallmann syndrome 3"[Title/Abstract] OR "Knobloch syndrome"[Title/Abstract] OR "Koolen-De Vries Syndrome"[Title/Abstract] OR "Lacrimoauriculodentodigital syndrome"[Title/Abstract] OR "Lathosterolosis"[Title/Abstract] OR "Laurence-Moon syndrome"[Title/Abstract] OR "Liddle syndrome"[Title/Abstract] OR "Lipoid adrenal hyperplasia"[Title/Abstract] OR "Loeys-Dietz syndrome 3"[Title/Abstract] OR "Lysinuric protein intolerance"[Title/Abstract] OR "Macrocephaly alopecia cutis laxa and scoliosis"[Title/Abstract] OR "Marden-Walker Syndrome"[Title/Abstract] OR "McArdle Disease"[Title/Abstract] OR "Medullary cystic kidney disease 1"[Title/Abstract] OR "Methylmalonic aciduria and homocystinuria cblc type"[Title/Abstract] OR "Methylmalonic aciduria and homocystinuria cblF type"[Title/Abstract] OR "Mevalonic aciduria"[Title/Abstract] OR "Micro syndrome"[Title/Abstract] OR "Microcephalic osteodysplastic primordial dwarfism type I"[Title/Abstract])) OR ("Autosomal dominant polycystic kidney disease"[Title/Abstract] OR "Autosomal dominant tubulointerstitial kidney disease"[Title/Abstract] OR "Autosomal recessive polycystic kidney disease"[Title/Abstract] OR "Bardet-Biedl syndrome"[Title/Abstract] OR "Cranioectodermal dysplasia"[Title/Abstract] OR "Ellis Van Creveld syndrome"[Title/Abstract] OR "Karyomegalic interstitial nephritis"[Title/Abstract] OR "Meckel syndrome"[Title/Abstract] OR "Nephronophthisis"[Title/Abstract] OR "Renal-hepatic-pancreatic dysplasia"[Title/Abstract] OR "RHYNS syndrome"[Title/Abstract] OR "Saldino-Mainzer syndrome"[Title/Abstract] OR "Senior-Loken syndrome"[Title/Abstract] OR "Tuberous sclerosis complex"[Title/Abstract] OR "Von Hippel-Lindau disease"[Title/Abstract] OR "Alport syndrome"[Title/Abstract] OR "HANAC syndrome"[Title/Abstract] OR "Action myoclonus-renal failure syndrome"[Title/Abstract] OR "Denys-Drash syndrome"[Title/Abstract] OR "Frasier syndrome"[Title/Abstract] OR "Galloway-Mowat syndrome"[Title/Abstract] OR "MYH9-related disease"[Title/Abstract] OR "Nail-patella-like renal disease"[Title/Abstract] OR "Nail-patella syndrome"[Title/Abstract] OR "Pierson syndrome"[Title/Abstract] OR "Schimke immuno-osseous dysplasia"[Title/Abstract] OR "Genetic nephrotic syndrome"[Title/Abstract] OR "Congenital nephrotic syndrome"[Title/Abstract] OR "Finnish type"[Title/Abstract] OR "Genetic steroid-resistant nephrotic syndrome"[Title/Abstract] OR "Idiopathic nephrotic syndrome"[Title/Abstract] OR "Primary membranoproliferative glomerulonephritis"[Title/Abstract] OR "C3 glomerulopathy"[Title/Abstract] OR "C3 glomerulonephritis"[Title/Abstract] OR "Dense deposit disease"[Title/Abstract] OR "Immunoglobulin-mediated membranoproliferative glomerulonephritis"[Title/Abstract] OR "Primary membranous glomerulonephritis"[Title/Abstract] OR "Familial Mediterranean fever"[Title/Abstract] OR "Fibronectin glomerulopathy"[Title/Abstract] OR "AApoAI amyloidosis"[Title/Abstract] OR "AApoAII amyloidosis"[Title/Abstract] OR "AFib amyloidosis"[Title/Abstract] OR "ALys amyloidosis"[Title/Abstract] OR "Hypocomplementemic urticarial vasculitis"[Title/Abstract] OR "Lipoprotein glomerulopathy"[Title/Abstract] OR "Muckle-Wells syndrome"[Title/Abstract] OR "AA amyloidosis"[Title/Abstract] OR "AApoAIV amyloidosis"[Title/Abstract] OR "Adult-onset Still disease"[Title/Abstract] OR "AH amyloidosis"[Title/Abstract] OR "AL amyloidosis"[Title/Abstract] OR "Behçet disease"[Title/Abstract] OR "Collagen type III glomerulopathy"[Title/Abstract] OR "Dermatomyositis"[Title/Abstract] OR "IgG4-related kidney disease"[Title/Abstract] OR "Immunotactoid"[Title/Abstract] OR "fibrillary glomerulopathy"[Title/Abstract] OR "Immunotactoid glomerulopathy"[Title/Abstract] OR "Juvenile dermatomyositis"[Title/Abstract]		

	OR "Juvenile polymyositis"[Title/Abstract] OR "Mixed connective tissue disease"[Title/Abstract])) OR ("Heavy chain deposition disease"[Title/Abstract] OR "Light and heavy chain deposition disease"[Title/Abstract] OR "Polymyositis"[Title/Abstract] OR "Reynolds syndrome"[Title/Abstract] OR "Sarcoidosis"[Title/Abstract] OR "Systemic sclerosis"[Title/Abstract] OR "Diffuse cutaneous systemic sclerosis"[Title/Abstract] OR "Limited cutaneous systemic sclerosis"[Title/Abstract] OR "Limited systemic sclerosis"[Title/Abstract] OR "Anti-glomerular basement membrane disease"[Title/Abstract] OR "Buerger disease"[Title/Abstract] OR "Cryoglobulinemic vasculitis"[Title/Abstract] OR "Mixed cryoglobulinemia type II"[Title/Abstract] OR "Eosinophilic granulomatosis with polyangiitis"[Title/Abstract] OR "Giant cell arteritis"[Title/Abstract] OR "Granulomatosis with polyangiitis"[Title/Abstract] OR "Immunoglobulin A vasculitis"[Title/Abstract] OR "Microscopic polyangiitis"[Title/Abstract] OR "Pauci-immune glomerulonephritis"[Title/Abstract] OR "Pediatric systemic lupus erythematosus"[Title/Abstract] OR "Polyarteritis nodosa"[Title/Abstract] OR "Cutaneous polyarteritis nodosa"[Title/Abstract] OR "Systemic polyarteritis nodosa"[Title/Abstract] OR "Relapsing polychondritis"[Title/Abstract] OR "Systemic lupus erythematosus"[Title/Abstract] OR "Takayasu arteritis"[Title/Abstract] OR "Beta thalassemia"[Title/Abstract] OR "Beta thalassemia intermedia"[Title/Abstract] OR "Beta thalassemia major"[Title/Abstract] OR "Dominant beta thalassemia"[Title/Abstract] OR "Fanconi anemia"[Title/Abstract] OR "Sickle cell anemia"[Title/Abstract] OR "Beckwith Wiedemann syndrome"[Title/Abstract] OR "Birt-Hogg-Dube syndrome"[Title/Abstract] OR "Hereditary leiomyomatosis and renal cell cancer"[Title/Abstract] OR "Hereditary papillary renal cell carcinoma"[Title/Abstract] OR "Hyperparathyroidism- jaw tumor syndrome"[Title/Abstract] OR "Perlman syndrome"[Title/Abstract] OR "Tuberous sclerosis complex"[Title/Abstract] OR "Von Hippel-Lindau disease"[Title/Abstract] OR "WAGR syndrome"[Title/Abstract] OR "Adenine phosphoribosyltransferase deficiency"[Title/Abstract] OR "Alkaptonuria"[Title/Abstract] OR "Alpha-1-antitrypsin deficiency"[Title/Abstract] OR "ALG8-CDG"[Title/Abstract] OR "Musculocontractural Ehlers-Danlos syndrome"[Title/Abstract] OR "SLC35A1-CDG"[Title/Abstract] OR "Dent disease"[Title/Abstract] OR "Enamel-renal syndrome"[Title/Abstract] OR "Fabry disease"[Title/Abstract] OR "Familial renal glucosuria"[Title/Abstract] OR "Fanconi-Bickel syndrome"[Title/Abstract] OR "Galactosemia"[Title/Abstract] OR "Classic galactosemia"[Title/Abstract] OR "Galactokinase deficiency"[Title/Abstract] OR "Galactose epimerase deficiency"[Title/Abstract] OR "Hartnup disease"[Title/Abstract] OR "Hereditary fructose intolerance"[Title/Abstract] OR "Hereditary xanthinuria"[Title/Abstract] OR "Xanthinuria type I"[Title/Abstract] OR "Xanthinuria type II"[Title/Abstract] OR "Hypoxanthine-guanine phosphoribosyltransferase deficiency"[Title/Abstract] OR "Lesch-Nyhan syndrome"[Title/Abstract] OR "Imerslund-Grasbeck syndrome"[Title/Abstract] OR "Infantile nephropathic cystinosis"[Title/Abstract] OR "Juvenile nephropathic cystinosis"[Title/Abstract] OR "LCAT deficiency"[Title/Abstract] OR "Familial LCAT deficiency"[Title/Abstract] OR "Fish-eye disease"[Title/Abstract])) OR ("Lowe Syndrome"[Title/Abstract] OR "Lysinuric protein intolerance"[Title/Abstract] OR "Phosphoribosylpyrophosphate synthetase superactivity"[Title/Abstract] OR "Porphyria"[Title/Abstract] OR "Acute hepatic porphyria"[Title/Abstract] OR "Acute intermittent porphyria"[Title/Abstract] OR "Hereditary coproporphyria"[Title/Abstract] OR "Chronic hepatic porphyria"[Title/Abstract] OR "Hepatoerythropoietic porphyria"[Title/Abstract] OR "Porphyria cutanea tarda"[Title/Abstract] OR "Congenital erythropoietic porphyria"[Title/Abstract] OR "Primary hyperoxaluria type 1"[Title/Abstract] OR "Primary hyperoxaluria type 2"[Title/Abstract] OR "Primary hyperoxaluria type 3"[Title/Abstract] OR "Sialidosis type 2"[Title/Abstract] OR "Tyrosinemia type 1"[Title/Abstract] OR "Vitamin B12-responsive methylmalonic acidemia"[Title/Abstract] OR "Vitamin B12-unresponsive methylmalonic acidemia"[Title/Abstract] OR "Wilson disease"[Title/Abstract] OR "Zellweger syndrome"[Title/Abstract] OR "Congenital renal artery stenosis"[Title/Abstract] OR "Apparent mineralocorticoid excess"[Title/Abstract] OR "William syndrome"[Title/Abstract] OR "Pseudoxanthoma elasticum"[Title/Abstract] OR "Liddle syndrome"[Title/Abstract] OR "Familial Hyperaldosteronism"[Title/Abstract] OR "Neurofibromatosis type 1"[Title/Abstract] OR "Pseudohypoaldosteronism type 2"[Title/Abstract] OR "Williams	

	Syndrome"[Title/Abstract] OR "Alstrom syndrome"[Title/Abstract] OR "Bartter syndrome"[Title/Abstract] OR "Cystinuria type A"[Title/Abstract] OR "Cystinuria type B"[Title/Abstract] OR "EAST syndrome"[Title/Abstract] OR "Gitelman syndrome"[Title/Abstract] OR "Hereditary renal hypouricemia"[Title/Abstract] OR "Hypophosphatemic rickets"[Title/Abstract] OR "Autosomal dominant hypophosphatemic rickets"[Title/Abstract] OR "Autosomal recessive hypophosphatemic rickets"[Title/Abstract] OR "Dent disease"[Title/Abstract] OR "Hereditary hypophosphatemic rickets with hypercalciuria"[Title/Abstract] OR "X-linked hypophosphatemia"[Title/Abstract] OR "Hypotonia-cystinuria syndrome"[Title/Abstract] OR "Idiopathic hypercalciuria"[Title/Abstract] OR "Jeune syndrome"[Title/Abstract] OR "Nephrogenic Diabetes insipidus"[Title/Abstract] OR "Oculocerebrorenal syndrome of Lowe"[Title/Abstract] OR "Oncogenic osteomalacia"[Title/Abstract] OR "Primary hypomagnesemia with secondary hypocalcemia"[Title/Abstract] OR "Renal Tubular Acidosis"[Title/Abstract] OR "Proximal renal tubular acidosis"[Title/Abstract] OR "Distal renal tubular acidosis"[Title/Abstract] OR "Osteopetrosis with renal tubular acidosis"[Title/Abstract] OR "Pseudohypoaldosteronism"[Title/Abstract] OR "Tubulointerstitial nephritis and uveitis syndrome"[Title/Abstract] OR "Clear cell sarcoma of kidney"[Title/Abstract] OR "Congenital mesoblastic nephroma"[Title/Abstract] OR "Multiloculated renal cyst"[Title/Abstract] OR "Nephroblastoma"[Title/Abstract] OR "Renal cell carcinoma"[Title/Abstract] OR "Acquired cystic disease - associated renal cell carcinoma"[Title/Abstract])) OR ("Chromophobe renal cell carcinoma"[Title/Abstract] OR "Collecting duct carcinoma"[Title/Abstract] OR "MiT family translocation renal cell carcinoma"[Title/Abstract] OR "Papillary renal cell carcinoma"[Title/Abstract] OR "Renal medullary carcinoma"[Title/Abstract] OR "Tubulocystic renal cell carcinoma"[Title/Abstract] OR "Clear cell renal carcinoma"[Title/Abstract] OR "Clear cell papillary renal cell carcinoma"[Title/Abstract] OR "Multilocular cystic renal neoplasm of low malignant potential"[Title/Abstract] OR "Congenital hydronephrosis"[Title/Abstract] OR "Patent urachus"[Title/Abstract] OR "Urachal cyst"[Title/Abstract] OR "Urachal diverticulum"[Title/Abstract] OR "Urachal sinus"[Title/Abstract] OR "Duplication of urethra"[Title/Abstract] OR "Exstrophy-epispadias complex"[Title/Abstract] OR "Bladder exstrophy"[Title/Abstract] OR "Cloacal exstrophy"[Title/Abstract] OR "Isolated epispadias"[Title/Abstract] OR "Familial vesicoureteral reflux"[Title/Abstract] OR "Anterior urethral valve"[Title/Abstract] OR "Posterior urethral valve"[Title/Abstract] OR "Prune belly syndrome"[Title/Abstract] OR "Medullary sponge kidney"[Title/Abstract] OR "Megacystis-megaureter syndrome"[Title/Abstract] OR "Bilateral multicystic dysplastic kidney"[Title/Abstract] OR "Unilateral multicystic dysplastic kidney"[Title/Abstract] OR "Oligomeganephronia"[Title/Abstract] OR "Bilateral Renal agenesis"[Title/Abstract] OR "Unilateral Renal agenesis"[Title/Abstract] OR "Renal hypoplasia unilateral"[Title/Abstract] OR "22q11.2 deletion syndrome"[Title/Abstract] OR "Di George syndrome"[Title/Abstract] OR "8q24.3 microdeletion syndrome"[Title/Abstract] OR "Acrorenal syndrome"[Title/Abstract] OR "AREDYLD syndrome"[Title/Abstract] OR "Arthrogryposis-renal dysfunction-cholestasis syndrome"[Title/Abstract] OR "Axial mesodermal dysplasia spectrum"[Title/Abstract] OR "BNAR syndrome"[Title/Abstract] OR "BOR syndrome"[Title/Abstract] OR "Cat-eye syndrome"[Title/Abstract] OR "Caudal regression syndrome"[Title/Abstract] OR "CHARGE syndrome"[Title/Abstract] OR "Cornelia de Lange syndrome"[Title/Abstract] OR "EEC syndrome"[Title/Abstract] OR "Ellis Van Creveld syndrome"[Title/Abstract] OR "Faciocardiorenal syndrome"[Title/Abstract] OR "Fraser syndrome"[Title/Abstract] OR "Hajdu-Cheney syndrome"[Title/Abstract] OR Hypoparathyroidism-sensorineural deafness-renal disease syndrome[Title/Abstract] OR "Jeune syndrome"[Title/Abstract] OR "Kabuki Syndrome"[Title/Abstract] OR "Kallmann syndrome"[Title/Abstract] OR "Meckel syndrome"[Title/Abstract] OR "Megacystis-microcolon-intestinal hypoperistalsis syndrome"[Title/Abstract] OR "Menke-Hennekam syndrome"[Title/Abstract] OR "Neurofaciodigitorenal syndrome"[Title/Abstract] OR "Noonan syndrome"[Title/Abstract] OR "Ochoa syndrome"[Title/Abstract] OR "Orofaciodigital syndrome type 1"[Title/Abstract] OR "Pallister-Hall syndrome"[Title/Abstract] OR "Renal coloboma syndrome"[Title/Abstract] OR "Renal nutcracker syndrome"[Title/Abstract] OR "Schinzel-Giedion syndrome"[Title/Abstract] OR "Simpson-Golabi-Behmel syndrome"[Title/Abstract]	

	OR "Smith-Lemli-Opitz syndrome"[Title/Abstract] OR "Thomas syndrome"[Title/Abstract] OR "Townes-Brocks syndrome"[Title/Abstract] OR "Trisomy 13"[Title/Abstract] OR "Trisomy 18"[Title/Abstract] OR "Turner syndrome"[Title/Abstract] OR "Monosomy X"[Title/Abstract] OR "Mosaic monosomy X"[Title/Abstract] OR "Ulbright-Hodes syndrome"[Title/Abstract] OR "VACTERL/VATER association"[Title/Abstract] OR "WAGR syndrome"[Title/Abstract] OR "Shiga toxin-associated hemolytic uremic syndrome"[Title/Abstract] OR "Streptococcus pneumoniae-associated hemolytic uremic syndrome"[Title/Abstract] OR "Pediatric systemic lupus erythematosus"[Title/Abstract] OR "Thrombotic thrombocytopenic purpura"[Title/Abstract] OR "Congenital thrombotic thrombocytopenic purpura"[Title/Abstract] OR "Immune-mediated thrombotic thrombocytopenic purpura"[Title/Abstract] OR "Acquired thrombotic thrombocytopenic purpura"[Title/Abstract] OR "Coenzyme q10 deficiency"[Title/Abstract] OR "Coffin Siris syndrome"[Title/Abstract] OR "Cockayne syndrome"[Title/Abstract])) OR ("Chromophobe renal cell carcinoma"[Title/Abstract] OR "Collecting duct carcinoma"[Title/Abstract] OR "MiT family translocation renal cell carcinoma"[Title/Abstract] OR "Papillary renal cell carcinoma"[Title/Abstract] OR "Renal medullary carcinoma"[Title/Abstract] OR "Tubulocystic renal cell carcinoma"[Title/Abstract] OR "Clear cell renal carcinoma"[Title/Abstract] OR "Clear cell papillary renal cell carcinoma"[Title/Abstract] OR "Multilocular cystic renal neoplasm of low malignant potential"[Title/Abstract] OR "Congenital hydronephrosis"[Title/Abstract] OR "Patent urachus"[Title/Abstract] OR "Urachal cyst"[Title/Abstract] OR "Urachal diverticulum"[Title/Abstract] OR "Urachal sinus"[Title/Abstract] OR "Duplication of urethra"[Title/Abstract] OR "Exstrophy-epispadias complex"[Title/Abstract] OR "Bladder exstrophy"[Title/Abstract] OR "Cloacal exstrophy"[Title/Abstract] OR "Isolated epispadias"[Title/Abstract] OR "Familial vesicoureteral reflux"[Title/Abstract] OR "Anterior urethral valve"[Title/Abstract] OR "Posterior urethral valve"[Title/Abstract] OR "Prune belly syndrome"[Title/Abstract] OR "Medullary sponge kidney"[Title/Abstract] OR "Megacystis-megaureter syndrome"[Title/Abstract] OR "Bilateral multicystic dysplastic kidney"[Title/Abstract] OR "Unilateral multicystic dysplastic kidney"[Title/Abstract] OR "Oligomeganephronia"[Title/Abstract] OR "Bilateral Renal agenesis"[Title/Abstract] OR "Unilateral Renal agenesis"[Title/Abstract] OR "Renal hypoplasia unilateral"[Title/Abstract] OR "22q11.2 deletion syndrome"[Title/Abstract] OR "Di George syndrome"[Title/Abstract] OR "8q24.3 microdeletion syndrome"[Title/Abstract] OR "Acrorenal syndrome"[Title/Abstract] OR "AREDYLD syndrome"[Title/Abstract] OR "Arthrogryposis-renal dysfunction-cholestasis syndrome"[Title/Abstract] OR "Axial mesodermal dysplasia spectrum"[Title/Abstract] OR "BNAR syndrome"[Title/Abstract] OR "BOR syndrome"[Title/Abstract] OR "Cat-eye syndrome"[Title/Abstract] OR "Caudal regression syndrome"[Title/Abstract] OR "CHARGE syndrome"[Title/Abstract] OR "Cornelia de Lange syndrome"[Title/Abstract] OR "EEC syndrome"[Title/Abstract] OR "Ellis Van Creveld syndrome"[Title/Abstract] OR "Faciocardiorenal syndrome"[Title/Abstract] OR "Fraser syndrome"[Title/Abstract] OR "Hajdu-Cheney syndrome"[Title/Abstract] OR Hypoparathyroidism-sensorineural deafness-renal disease syndrome[Title/Abstract] OR "Jeune syndrome"[Title/Abstract] OR "Kabuki Syndrome"[Title/Abstract] OR "Kallmann syndrome"[Title/Abstract] OR "Meckel syndrome"[Title/Abstract] OR "Megacystis-microcolon-intestinal hypoperistalsis syndrome"[Title/Abstract] OR "Menke-Hennekam syndrome"[Title/Abstract] OR "Neurofaciodigitorenal syndrome"[Title/Abstract] OR "Noonan syndrome"[Title/Abstract] OR "Ochoa syndrome"[Title/Abstract] OR "Orofaciodigital syndrome type 1"[Title/Abstract] OR "Pallister-Hall syndrome"[Title/Abstract] OR "Renal coloboma syndrome"[Title/Abstract] OR "Renal nutcracker syndrome"[Title/Abstract] OR "Schinzel-Giedion syndrome"[Title/Abstract] OR "Simpson-Golabi-Behmel syndrome"[Title/Abstract] OR "Smith-Lemli-Opitz syndrome"[Title/Abstract] OR "Thomas syndrome"[Title/Abstract] OR "Townes-Brocks syndrome"[Title/Abstract] OR "Trisomy 13"[Title/Abstract] OR "Trisomy 18"[Title/Abstract] OR "Turner syndrome"[Title/Abstract] OR "Monosomy X"[Title/Abstract] OR "Mosaic monosomy X"[Title/Abstract] OR "Ulbright-Hodes syndrome"[Title/Abstract] OR "VACTERL/VATER association"[Title/Abstract] OR "WAGR syndrome"[Title/Abstract] OR "Shiga toxin-associated hemolytic uremic syndrome"[Title/Abstract] OR "Streptococcus pneumoniae-associated hemolytic uremic syndrome"[Title/Abstract] OR "Pediatric	

	systemic lupus erythematosus"[Title/Abstract] OR "Thrombotic thrombocytopenic purpura"[Title/Abstract] OR "Congenital thrombotic thrombocytopenic purpura"[Title/Abstract] OR "Immune-mediated thrombotic thrombocytopenic purpura"[Title/Abstract] OR "Acquired thrombotic thrombocytopenic purpura"[Title/Abstract] OR "Coenzyme q10 deficiency"[Title/Abstract] OR "Coffin Siris syndrome"[Title/Abstract] OR "Cockayne syndrome"[Title/Abstract])) OR ("Cardiofaciocutaneous syndrome"[Title/Abstract] OR "Burn-McKeown syndrome"[Title/Abstract] OR "Blackfan-Diamond anemia"[Title/Abstract] OR "Adrenal hypoplasia"[Title/Abstract] OR "Alkaptonuria"[Title/Abstract] OR "Acro-renal-ocular syndrome"[Title/Abstract] OR "5-oxoprolinase deficiency"[Title/Abstract] OR "Autosomal dominant periodic fever syndrome"[Title/Abstract] OR "Hereditary hemorrhagic telangiectasia"[Title/Abstract] OR "Hereditary orotic aciduria"[Title/Abstract] OR "Hermansky-Pudlak syndrome 1"[Title/Abstract] OR "Hydrolethalus syndrome"[Title/Abstract] OR "Hydroxykynureninuria"[Title/Abstract] OR "Hypercalcemia infantile 1"[Title/Abstract] OR "Hyperglycinuria"[Title/Abstract] OR "GM1-gangliosidosis type 1"[Title/Abstract] OR "GM1-gangliosidosis type 2"[Title/Abstract] OR "Hand-foot-uterus syndrome"[Title/Abstract] OR "Hennekam Lymphangiectasia-Lymphedema Syndrome"[Title/Abstract] OR "Hereditary angiopathy with nephropathy aneurysms and Muscle cramps"[Title/Abstract] OR "Glomerulopathy with fibronectin deposits 2"[Title/Abstract] OR "Glutaric aciduria 3"[Title/Abstract] OR "Galloway-Mowat syndrome"[Title/Abstract] OR "Gaucher disease"[Title/Abstract] OR "Genitopatellar syndrome"[Title/Abstract] OR "Fanconi anemia complementation group a"[Title/Abstract] OR "Fanconi anemia complementation group b"[Title/Abstract] OR "Fanconi anemia complementation group c"[Title/Abstract] OR "Fanconi anemia Complementation group d2"[Title/Abstract] OR "Fanconi anemia complementation group e"[Title/Abstract] OR "Fanconi anemia complementation group I"[Title/Abstract] OR "Fanconi anemia complementation group L"[Title/Abstract] OR "Cockayne syndrome"[Title/Abstract] OR "Feingold syndrome"[Title/Abstract] OR "Floating-Harbor syndrome"[Title/Abstract] OR "Focal dermal hypoplasia"[Title/Abstract] OR "Diaphanospondylodysostosis"[Title/Abstract] OR "Donnai-barrow syndrome"[Title/Abstract] OR "DOOR syndrome"[Title/Abstract] OR "Dyskeratosis congenita"[Title/Abstract]))	
#3	#1 AND #2	1239
#4	#3 and Limit by last 10 years (from 2013 – 2023)	824

Figure S1 - PRISMA flow chart of study inclusion

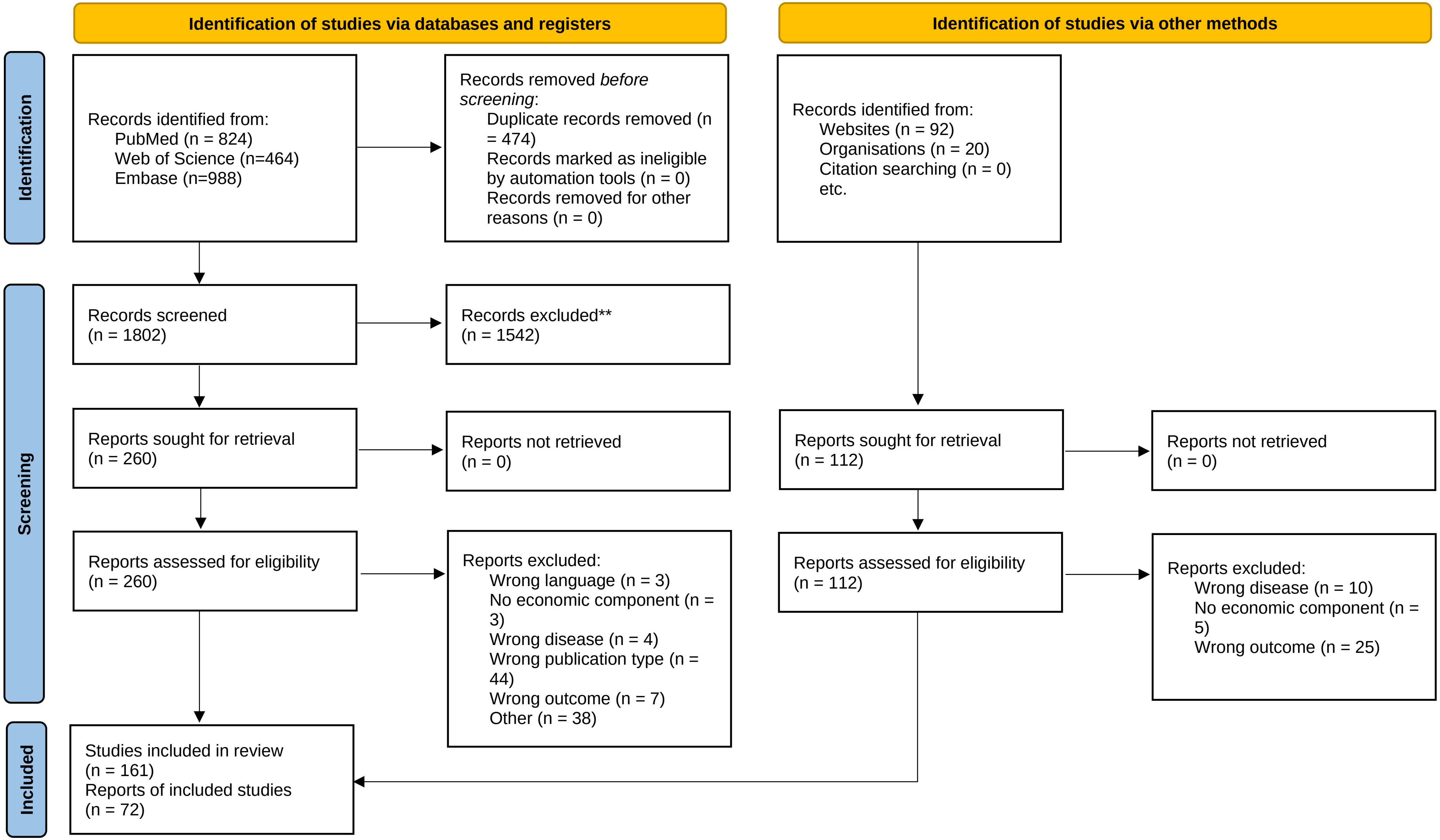

Table S2 – Overview of cost-of-illness studies

Author	Year	Country	RKD	Perspective	Study period	Sample size	Cost elements included	Result	Key drivers of costs	Funding
Jonsen	2016	Sweden	Systemic Lupus Erythematosus	Societal	8 years	1,029 SLE patients	Direct medical costs (inpatient, specialist outpatient and drugs) and indirect costs (sickness leave and disability pensions)	The total annual cost per patient was 208,555 SEK ($33,369) of which the direct costs were 63,672kr ($10,188) and indirect costs were 144,883 SEK ($23,181)	Costs (direct and indirect) increased for patients with short disease duration, higher disease activity and those with damage in the neuropsychiatric and musculoskeletal domains. Organ damage in the renal and ocular systems increased direct costs	Glaxo Smith Kline
Kim	2020	South Korea	Systemic Lupus Erythematosus and systemic sclerosis	Health care payer	5 years	19,441 SLE patients, 3,606 systemic sclerosis patients	Direct medical costs	Annual medical costs per patient were: $1425 in 2016 for SLE patients and $1,440 for SSc patients.	For both SLE and SSc patients, medications accounted for the majority of costs, however, lab tests grow at the fastest rate between 2012-2016, accounting for a larger portion of total costs.	Rheumatology Research Foundation of South Korea

Connolly	2021	Belgium	Acute hepatic porphyria	Societal	Lifetime model	NA	Medical costs, disability payment, tax intake	Lifetime health costs were estimated to be over 3 million Euros. Individuals with Acute Hepatic porphyria had reduced lifetime earnings (347,802 Euros less), resulting in tax intake €183,187 lower than general population. There was an associated increase in disability payments of €179,184 per person over a lifetime.	Frequent hospitalisations from porphyria attacks were the key drivers of cost	Alnylam Pharmaceutical, The Netherlands.
Anandarajah	2017	USA	Systemic lupus erythematosus	Patients	2 years	104	Direct medical costs and indirect costs (lost productivity)	The mean annual cost per patient for hospitalization was US$51,808.	Hospitalisation, length of stay and readmission	No funding source reported
Bell	2022	USA	Systemic lupus erythematosus	Health payer	10 years	8952	Direct medical costs including inpatient, outpatient, pharmaceuticals	Inpatient, emergency department and outpatient claims increased from pre- to post-index date and mean (SD) all-	Direct medical costs for treating organ damage in patients with SLE	Funded by GlaxoSmithKline

								cause costs were 71% higher post- versus pre-index date		
Bell	2023	USA	Systemic lupus erythematosus with and without lupus nephritis	Health payer	2 years	11,663 (LN, n = 2,916; non-LN, n = 8,747)	Direct medical costs including inpatient, outpatient, pharmaceuticals	The mean total healthcare costs associated with SLE flares of any severity were greater for patients with LN	Patients with SLE + LN experience more frequent and severe disease flares resulting in higher direct medical costs	Funded by GlaxoSmithKline
Bell	2023	USA	Systemic lupus erythematosus with and without lupus nephritis	Health payer	1 year	2,310 patients with LN and 2310 matched patients who had SLE without LN	Direct medical costs including inpatient, outpatient, pharmaceuticals	Total all-cause costs per patient in the LN cohort were also significantly higher compared with the SLE without LN cohort	All-cause healthcare resource utilization was higher for SLE patients with LN resulting in significantly higher costs	Funded by GlaxoSmithKline
Bell	2021	USA	Eosinophilic granulomatosis with polyangiitis (EGPA)	Health payer	10 years	8,904 patients matched (EGPA (n = 2,226), asthma (n = 6,678))	Direct medical costs including inpatient, outpatient, pharmaceuticals	Patients with EGPA have more comorbidities, greater health care costs and HCRU, and use SCS more frequently than patients with asthma	Patients with EGPA experience greater costs due to higher health care resource utilisation and systemic corticosteroid use	Funded by GlaxoSmithKline

Betts	2020	USA	Epilepsy and Tuberous sclerosis complex	Health payer	1 year	2,028 patients with epilepsy + TSC matched to 10,140 patients with epilepsy alone	Direct medical costs including inpatient, outpatient, pharmaceuticals	Epilepsy-related costs were significantly higher for patients with TSC; the cost difference per patient was $14,639 for prescription costs ($p < 0.001$), and the charge difference per patient was $16,838 for medical charges	Higher utilisation of healthcare services, possibly including long-term care facilities, as well as surveillance with frequent diagnostic investigations	Supported by Greenwich Biosciences, Inc.
Bhandari	2022	USA	Renal cell carcinoma	Health payer	8 years	11,228 RCC patients matched with 56,140 controls	Direct medical costs including inpatient, outpatient, pharmaceuticals	Per-patient cumulative average total costs were 3.6 and 1.7 times higher for RCC vs controls, at 1 and 5 years respectively.	Prescription drug costs accounted for 8.4% and 18.1% of the 1-year and 5-year incremental total costs, respectively. RCC patients had greater cumulative number of hospitalizations, emergency department visits and prescriptions	No funding source reported

Blaylock	2020	USA	Acute intermittent porphyria	Health payer	2 years	139	Direct medical costs including inpatient, outpatient, pharmaceuticals	Real-world annualized total expenditures for all-cause (any diagnosis) and AIP-related care are much less than previous estimates of AIP treatment costs.	NA	Funded by Recordati Rare Diseases.
Buja	2022	Italy	Renal cell carcinoma	Health payer	2 years	148	Direct medical costs including inpatient, outpatient, pharmaceuticals	Two years after diagnosis, the average total costs amounted to €21,429 per patient. Expenditure for stage IV patients was 2.44 times higher than for stage I patients.	Hospitalization appeared to be the most expensive item for both early and advanced disease. In the second year, however, outpatient procedures were the main cost driver in the earlier stages, whereas anticancer drugs accounted for the highest costs in the advanced stages	No funding source reported

Chiu	2022	Taiwan	Systemic lupus erythematosus	Health payer	4 years	22,258	Direct medical costs including inpatient, outpatient, pharmaceuticals	The estimated annual expenditures incurred by SLE patients without organ damage accrual were US$3,167, while the estimated annual expenditure incurred by whole cohort of SLE patients in the first 6 months was US$5094	Medical costs associated with organ damage, particularly the ocular and musculoskeletal systems (incurring annual medical costs of US$4615 and US$19,363, respectively).	Funded by GlaxoSmithKline
Cho	2014	South Korea	Systemic lupus erythematosus	Societal	2 months	201	Direct medical costs; direct nonmedical costs; indirect costs representing productivity loss	The average total cost of illness was estimated to be KRW 9.82 million (US $ 8,993) per year, of which 41.6% was accounted for by direct costs and 58.4% by indirect costs.	The occurrence of a renal disorder and depression were both associated with increased annual total and direct costs	Supported by the MKE/KEIT R&D Program the Ministry of Health and Welfare, Republic of Korea.

Cholley et al.	2018	France	Metastic renal cell carcinoma	Health payer	NA	224	Direct medical costs including travel time	The mean cost of illness was estimated at 71,185 (plus/minus 52,683). Outpatient/inpatient treatment and hospitalization represented 76.0% and 19.7% of this cost, respectively	Cost of illness was significantly associated with time of disease control for metastatic first-line treatment of greater than 6 months, more than 2 lines of treatment, nephrectomy at metastatic stage, lack of metastases at presentation, being younger than 65 at presentation	No funding source reported
Chu	2020	Hong Kong	Tuberous sclerosis complex	Health payer	10 years	304	Direct medical costs including inpatient and outpatient	Total average cost per patient per year was US$7,701	Out patient visits accounted for 75.6% of total average cost per patient per year, followed by ICU (18.4%)	No funding received
Clarke	2015	Canada	Systemic lupus erythematosus	Health payer	2 years	109	Direct medical costs including inpatient, outpatient, pharmaceuticals	The average annual direct medical cost was $10,608 Canadian (2010 dollars) and was higher for patients with severe disease, $15,048 versus $5,917 for mild	Cost was driven by inpatient stays and medication.	Funded by GlaxoSmithKline

Clarke	2020	USA	Systemic lupus erythematosus	Health payer	1 year	9033	Direct medical costs including inpatient, outpatient, pharmaceuticals	Adjusted mean total healthcare costs, excluding pharmacy, for moderate/severe SLE patients were higher than for mild SLE patients in the commercially-insured ($39,021 versus $23,519; p<0.0001) and Medicaid-insured populations ($56,050 versus $44,932; p=0.06).	For Medicaid-insured patients, inpatient admission costs were the largest single driver of costs. For commercially-insured patients, outpatients costs were.	Funded by AstraZeneca
Dall'Era	2023	USA	Lupus nephritis	Health payer	12 years	21,251	Direct medical costs including inpatient, outpatient, pharmaceuticals	Mean medical costs were $4,777 per month in active disease and $18,084 per month in ESRD (end-stage renal disease) vs $2,523 per month in low disease activity	The main medical cost drivers were inpatient visits ($13,756) and other outpatient visits ($3,013).	Funded by Aurinia Pharmaceuticals

Doria	2015	Italy	Systemic lupus erythematosus	Health payer	2 years	96	Direct medical costs including inpatient, outpatient, pharmaceuticals	The annual medical cost was 1.6 times higher in severe than in non-severe patients (€2,101 vs. €1,320; p=0.031). The cost of medications was also 2.5 times higher in severe patients (€1101 vs. €445, p=0.007).	Medications, especially immunosuppressant drugs, were the major cost drivers since they represented the large majority of the total cost of SLE.	Funded by GlaxoSmithKline
D'Souza	2022	USA	Amyloid light chain amyloidosis	Health payer	3 years	1,341 admissions	Direct medical costs	The mean (SD) total hospitalization cost was $27,099 ($34,849) for hospitalized patients with AL amyloidosis, compared to an the average cost of $14,661 ($129) for all hospital stays.	Diagnostic hospitalizations had higher cost ($40,052 vs $24,360; p < 0.001) and higher total charges ($161,526 vs $104,129; p < 0.001) than nondiagnostic ones	Funded by Prothena Biosciences Ltd.

Eriksson	2017	Multi-country (Denmark, Finland, Norway and Sweden)	Autosomal dominant polycystic kidney disease	Patients	1 year	243	Direct medical costs and indirect costs including informal care and productivity loss (using the human capital approach).	Average total annual costs were highest for dialysis patients, followed by transplant recipients, patients in CKD stages 4–5 and CKD stages 1–3 (P < 0.0001, for all countries). Compared to CKD stages 1–3, annual costs were almost twice as high in CKD stages 4–5, two to three times higher in transplant recipients, and seven to nine times higher in dialysis patients.	Direct costs were almost twice as high in patients with CKD stages 4–5 compared to stages 1–3, but around six times higher among transplant recipients and 21 times higher among dialysis patients.	Funded by Otsuka Pharma Scandinavia

Fagnani	2022	France	Tuberous sclerosis complex with epilepsy and tuberous sclerosis complex without epilepsy	Health payer	13 years	3,139	Direct medical costs including inpatient, outpatient, pharmaceuticals	Patients with epilepsy had higher annual healthcare costs (both inpatient and outpatient costs) compared with patients without epilepsy. A greater proportion of patients with epilepsy than without epilepsy were hospitalized (46% vs 32%) or visited a hospital specialist (65% vs 51%) at least once in 2018.	The most notable cots difference was seen in medication costs, which were 2.8 times higher in patients with epilepsy than those without.	Funded Jazz Pharmaceuticals, Inc.
Fatoye et al	2021	Canada	Systemic lupus erythematosus	Health payer	1 year	10,932 SLE cases (M = 2,546; F = 8,386). 41,851,36 non-SLE controls (M = 21,157,76; F = 20,693,60).	Direct medical costs	The mean annual costs of SLE, and non-SLE per case were $7,740.19 (Male = $7,986.59; Female = $7,665.38), and $2,479.53 (Male = $2,265.57; Female = $2,698.30), (p < 0.001) respectively.	The key cost drivers for the direct health care costs of SLE were inpatient hospital services (49%).	No funding received

Furst et al	2013	USA	Systemic lupus erythematosus	Health payer	1 year	907 (88% F) patients with SLE+LN. 1,062 (93% F) patients with NPSLE	Direct medical costs	Average annual healthcare costs for subjects with LN were $33,472 (95% CI = $29,797–$37,146). For NPSLE subjects, average annual healthcare costs were $30,341 (95% CI = $27,209–$33,474). Both are significantly higher (by 6–7-fold) compared to controls.	Patients with NPSLE had higher mean post-index numbers of ambulatory visits, specialist visits, emergency department visits and inpatient hospital stays, compared to controls (all p < 0.001).	Sponsored by MedImmune, LLC.
Gagnon-Sanschagrin	2021	USA	Autosomal dominant polycystic kidney disease	Health payer	1 year	9,227 cases	Direct medical costs	A mean cost difference of $22,879 per patient per year between ADPKD cases and controls	The largest incremental costs were observed in the end-stage renal disease requiring renal replacement therapy, driven by the cost of dialysis	Funded by Otsuka Pharmaceutical Development & Commercialization, Inc.
Geynisman et al	2014	USA	Metastic renal cell carcinoma	Health payer	1 year	767 patients receiving modern therapy < 65 years	Direct medical costs	First-year drug cost per patient with ancillary services = $59,664 for first-line oral treatment. $86,518 for	Drug costs increased significantly between 2004-2010, switching treatments	No funding source reported

								first-line IV treatment		
Hagiwara	2013	USA	Renal cell carcinoma	Health payer	1 year	881 patients aged > 65 years with adverse events	Direct medical costs excluding the costs of targeted therapy	Mean (SD) total cost of care over 30 days was substantially higher among patients with AEs ($13,944 [$14,529]) compared with those without mention of these events ($1878 [$5264]).	Treatment costs for adverse events	Funded by GlaxoSmithKline
Jiang et al.	2021	USA	Systemic lupus erythematosus	Health payer	2 years	2227	Direct medical costs including inpatient, outpatient, pharmaceuticals	Mean per-patient costs post-diagnosis for mild SLE: US$13,415; moderate SLE: US$29,512; severe SLE: US$68,260.	Leading mean cost drivers were outpatient visits (US$13 566) and hospitalisations (US$10 252).	Funded by AstraZeneca
Jonasch	2023	USA	von Hippel-Lindau disease-associated renal cell carcinoma	Health payer	13 years	160 with disease, 800 controls	Direct medical costs	Patients with the disease have $36,450 higher annual healthcare costs than patients without.	Annual cost difference was mainly driven by higher medial costs ($47,372 vs. $9,671 per year)	Funded by Merck & Co., Inc

Jönsen	2015	Sweden	Systemic lupus erythematosus	Societal	8 years	127 SLE patients and 508 population controls	Direct medical costs including inpatient, outpatient, pharmaceuticals	The mean annual total cost for SLE patients was SEK 180,520 ($30,093), compared to controls of SEK 59,985 ($10,000)	72% of total costs were due to indirect costs, 3% to SLE-specific pharmaceuticals, and the remaining 25% were in- and outpatient related costs.	Supported by a research grant from Glaxo SmithKline
Kan	2013	USA	Systemic lupus erythematosus	Health payer	7 years	14,262 SLE patients matched to controls	Direct medical costs including inpatient, outpatient, pharmaceuticals	The incremental annual cost associated with SLE was $10,984. Cost per flare was $11,716 for severe flares, $562 for moderate flares, and $129 for mild flares	Inpatient and outpatient costs were the dominant cost drivers, consisting of 47% and 38% of total cost for SLE patients.	Funded by GlaxoSmithKline and Human Genome Sciences
Karl	2017	Germany	Alpha-1 Antitrypsin Deficiency associated with chronic obstructive pulmonary disease	Patients	2 years	131 AATD patients (106 with, 25 without augmentation therapy (AT)) and 2,049 COPD patients	Direct medical costs and indirect costs	Adjusted mean direct annual costs were €6,099 in AATD patients without AT, €7,117 in AATD patients with AT, and €7,460 in COPD patients without AATD. There were no significant differences between groups	Not reported	Study was supported by the Competence Network Asthma and COPD (ASCONET)

								regarding indirect costs.		
Kawalec	2015	Poland	Systemic sclerosis, Systemic lupus erythematosus and sarcoidosis	Societal	1 year	4,800 (SLE: 1600; SS: 500; sarcoidosis: 2700)	Indirect costs due to absenteeism, based on the human capital approach	For SLE, systemic sclerosis and sarcoidosis in 2012, total indirect costs were as high as 7,260,595, 2,268,571 and 4,027,575 EUR, respectively.	For SLE and systemic sclerosis, long-term disability is the main cost driver (74.2% and 76.7% of costs, respectively). For sarcoidosis, sick leave accounted for 53.9% of costs.	No funding source reported
Khamashta	2013	UK	Systemic lupus erythematosus	Health payer	2 years	86 patients, 38 with severe SLE and 48 non-severe SLE	Direct medical costs	The mean annual direct medical cost of SLE was estimated at €3,231 per patient and was 2.2 times higher in patients with severe SLE compared with patients with non-severe SLE	Renal disease involvement and severe flares were associated with higher annual direct costs	Funded by GlaxoSmithKline

Kingswood	2016	UK	Tuberous sclerosis complex	Health payer	3 years	286 patients with TSC matched to controls	Direct medical costs including inpatient, outpatient, pharmaceuticals	TSC patients had costs totaling £12,681 per patient over the 3-year period, a figure 2.7-fold greater than the total costs in the comparator cohort (£4,777).	Manifestation in other organs (particularly, kidney, urinary tract and nervous system) significantly increased patient costs.	Funded by Novartis
Knarborg	2022	Denmark	Systemic sclerosis with and without interstitial lung disease (ILD)	Societal	9 years	1,869 cases and 7463 matched controls	Direct medical costs and indirect costs (estimated as the difference in earnings between cases and controls based on earned income and various social security compensation)	Total excess cost (direct healthcare, elderly care and indirect costs) in the systemic sclerosis-ILD cohort was €29,725, and €17,905 in the non-ILD systemic sclerosis cohort	Healthcare costs and forgone earnings were the key drivers of excess costs in both cohorts.	Funded by Boehringer Ingelheim
Liu	2017	China	Renal cell carcinoma	Health payer	5 years	NA	Direct medical costs including inpatient, outpatient, pharmaceuticals	Drug costs accounted for the largest proportion of medical expenditures each year, with the highest of 37.8% in 2012,	Drug costs shared the largest proportion (40.95%) of structural variation, followed by the costs of	Supported by Beijing Municipal Commission of Science and Technology

								slowly declining in following years.	surgeries (18.35%).	
Lokhandwala	2021	USA	Systemic lupus erythematosus patients initiating intravenous belimumab	Health payer	2 years	908 (female, 93.4%)	Direct medical costs including inpatient, outpatient, pharmaceuticals	Despite reduced disease activity, mean all-cause hospital-based costs increased 1.4 from the pre-index to the post-index period ($7,735 vs $11,030; p = 0.396)	Drug acquisition cost and administration schedule for IV belimumab	Funded by GlaxoSmithKline
Lopez-Bastida et al	2014	Spain	Systemic sclerosis	Societal	1 year	147	Direct medical costs, direct non-health care costs (e.g., informal care) and loss of labour productivity (using the human capital based approach)	The average annual cost per SS patient was €21,042. Direct health care costs amounted to €8,235, direct non–health care costs were €5,503, and loss of labor productivity was €7,303.	The largest expenditures for SS patients were early retirement, informal care, and medication.	Supported by the European Union

Maroun	2016	France	Renal cell carcinoma	Health payer	5 years	15,752 (female, 32%)	Direct medical costs including inpatient, outpatient, pharmaceuticals	The mean annual costs per patient for outpatient hospitalisations and inpatient hospitalisations were respectively 7,413€ (SD: 15,679) and 12,259€ (SD: 11,348).	Inpatient hospitalisations and expensive drugs	No funding source reported
McCormick et al.	2018	Canada	Systemic lupus erythematosus	Health payer	6 years	3,632 SLE cases (86% female) and matched 18,060 controls	Direct medical costs including inpatient, outpatient, pharmaceuticals	Per-person all-cause medical costs for SLE the year after diagnosis averaged C$12,019 (2013) compared to C$2,412 for non-SLE patients. Leading up to diagnosis, per-person healthcare costs for SLE patients increased year-over-year by 35%, on average.	The year after diagnosis, 58% of costs were due to hospitalisations, 24% outpatient, and 18% from prescription medications.	Supported by the Canadian Arthritis Networkand the Canadian Institutes of Health Research
Miyazaki	2020	Japan	Systemic lupus erythematosus	Health payer	6 years	4733 (78.5% female)	Direct medical costs including	Total annual per-patient cost = ¥1,017,012	Severity of disease	Funded by Janssen Pharmaceutical K.K

							inpatient, outpatient, pharmaceuticals			
Morrisroe	2017	Australia	Systemic sclerosis	Patients	4 years	531	Direct medical costs including inpatient, outpatient, pharmaceuticals	Total healthcare utilization cost to the Australian government extrapolated to all Australian SSc patients from 2011 to 2015 was AUD $297,663,404, an average annual cost of AUD $59,532,680 (US $43,816,040) and annual cost per patient of AUD$11,607 (US$8,542)	hospital related cost accounted for the majority of the total direct healthcare cost (44.4%), followed by medication cost (31.2%) and ambulatory care cost (21.1%).	No funding source reported
Neeleman	2018	Netherlands	Acute intermittent porphyria	Health payer	NA	88 (68.2% female) including 53 asymptomatic controls	Direct medical costs including inpatient, outpatient, pharmaceuticals	Total medical healthcare cost for recurrent cases group was €5.8 million versus €0.3 million for the symptomatic cases group	Not reported	No funding source reported

Padala	2022	New Zealand	Systemic sclerosis	Patients	6 months	86 (90% female)	Direct medical costs and productivity loss	The average total direct costs for the 6 months for health as reported by patients was NZ$ 444.50. The calculated percentage productivity loss was 46.5%, amounting to an average loss of NZ$ 22,786 annual income.	Mobility aids and protective gear accounted for 29% of costs, followed by self-funded health practitioner visits (23%).	No funding source reported
Park	2015	South Korea	Systemic lupus erythematosus	Health payer	1 year	749 (92.7% female)	Direct medical costs including inpatient admissions and outpatient visits	Overall annual direct medical costs amounted to USD 3,305 (2010 currency), of which 60.4% and 39.6% accounted for inpatients and outpatient costs, respectively.	The largest cost component was medication at 38.4% (USD 1269) followed by diagnostic procedures and tests (35.6%)	Funded by GlaxoSmithKline
Petri	2015	USA	Systemic lupus erythematosus	Health payer	6 years	1,721 pregnant women with SLE matched with 8605 controls	Direct medical costs including inpatient, outpatient, pharmaceuticals	The mean (SD) total all-cause direct healthcare costs were $21,509 ($24,438) for pregnant women compared to $11,481	Not reported	Sponsored by UCB Pharma

								($10,619; p < 0.0001) for controls.		
Pollissard	2021	USA	Thrombotic thrombocytopenic purpura	Health payer	8 years	2,279 (65.1% female)	Direct medical costs including out of pocket contributions	Total all-cause healthcare expenditures were significantly higher for patients with aTTP vs. without aTTP. Incremental cost of care varied by payer: Medicare Fee For Service: $7,124; Medicare Advantage: $5,918, commercial: $5,151, managed Medicaid: $5,431)	Across all payer types, hospitalizations were the major driver of healthcare expenditures	Sponsored by Sanofi
Poudel	2018	USA	Systemic sclerosis	Health payer	1 year	9,731 hospitalisations	Direct medical costs including inpatient, outpatient, pharmaceuticals	Median (interquartile range) cost was US$8,885 (-5,169, 15,921)	Higher cost of hospitalisation was predicted by the following factors: hospital from the West region, acute renal failure, acute bowel	No funding received

									obstruction and aspiration (aOR > 2.0 with P < 0.0001 for all).	
Prada	2021	Colombia	Systemic lupus erythematosus with and without lupus nephritis	Health payer	2 years	3,295 (90.1% female): 1,117 SLE patients with LN and 2,475 SLE patients without LN	Direct medical costs including inpatient, outpatient, pharmaceuticals	Average annual per-patient, all-claims, all-cause direct cost for lupus nephritis was US$ 12,624, 7.5 times higher than the average lupus patient without lupus nephritis.	The cost and frequency of procedures, and drug costs.	No funding source reported
Qi	2021	China	Gaucher Disease Type 1	Patients	NA	98: 49 patients (49% female) and 49 caregivers	Direct medical, direct non-medical, and indirect costs (including daily lost wages of caregivers)	Average annual economic burden of GD was estimated at $48,771. Indirect costs reflected an annual productivity loss of $1,980 per patient.	Direct healthcare costs of GD accounted for 86% of total costs, followed by medicines (61%) and inpatient treatment (15%)	Funded by The National Social Science Foundation of China
Quock	2018	USA	Amyloid light chain amyloidosis	Health payer	1 years	249 cases (41.8% female) matched to 747 controls	Direct medical costs including inpatient, outpatient, excluding	Mean (SD) healthcare costs were significantly higher for newly diagnosed AL amyloidosis patients than	Inpatient hospital costs ($28,126 [40,409]) and non-ED outpatient service costs	Funded by Prothena Biosciences Inc.

							pharmaceuticals	compared to controls ($71,040 [65,766] vs $13,722 [27,493)	($37,137 [37,363])	
Quock	2018	USA	Amyloid light chain amyloidosis	Health payer	9 years	7,326 (45.0% female)	Direct medical costs including inpatient, outpatient, pharmaceuticals	Mean (SD) total annual all-cause healthcare costs among all incident patients were $122,180 (159,074) in the year following diagnosis. Among incident patients with ≥24 months of follow-up, total mean (SD) costs decreased over time from $104,672 (134,993) in year 1 to $68,502 (121,711) in year 2.	In the year following diagnosis, medical (non-outpatient pharmacy) costs accounted for 90.5% of total costs.	Funded by Prothena Biosciences Inc.

Sieluk	2020	USA	Alpha-1 Antitrypsin Deficiency associated with chronic obstructive pulmonary disease	Health payer	6 years	953 cases matched with 7,928 controls	Direct medical costs including inpatient, outpatient, pharmaceuticals	The incremental cost difference between AATD-associated COPD patients and controls totaled $6861 [95% CI: $3025 - $10,698] and $5772 [95% CI: $1940 - $9604] per patient before and after the index date, respectively.	Cases incurred higher outpatient costs (cost ratio: 1.490), higher costs for other services (cost ratio: 11.834) and prescription drug costs (cost ratio: 1.372).	No funding source reported
Soerensen	2015	Denmark	Metastic renal cell carcinoma patients initiating targeted therapy	Health payer	2 years	631 (32% female)	Direct medical costs and lost productivity costs	Total health care costs per year were €5,348,352 in 2002–2005 and €12,149,764 in 2006–2009. Productivity costs (foregone earnings) were €7,852 in 2006–2009 and €8,265 in 2002–2005	Inpatient care was the main cost driver	Funded by unrestricted research grants
Strzelczyk	2021	Germany	Tuberous sclerosis complex patients with and without epilepsy	Health payer	10 years	256: 93 with epilepsy and 163 without	Direct medical costs including inpatient, outpatient, pharmaceuticals	For all patients, the mean annual cost of healthcare was €6,139 per patient-year. Patients with epilepsy incurred more than twice the	Healthcare costs were mostly attributable to medication (35%) and inpatient care (29%). Patients with epilepsy had greater	Funded by GW Pharmaceuticals

								mean annual healthcare costs of those without epilepsy (€9091 vs. €4583)	medication costs (€3819 vs. €1261 PPY)	
Sun	201 5	USA	Tuberous sclerosis complex patients following surgical resection of subependymal giant-cell astrocytoma (SEGA)	Health payer	2 years	47 (34% female)	Direct medical costs including inpatient, outpatient, pharmaceuti cals	Mean total direct medical costs per patient increased from $8,543 for the pre-surgery year to $85,397 for the post-surgery year.	The majority of the cost increase came from inpatient care ($67,792 or 88.2%) and outpatient care ($8024 or 10.4%).	Funded by Novartis
Sundaram	201 5	USA	Renal cell carcinoma post-nephrectomy	Health payer	2 years	643: 269 (35.3% female) with recurrence and 374 (42.2% female) without recurrence	Direct medical costs including inpatient, outpatient, pharmaceuti cals	Mean monthly total health care costs were $10,834 for patients with recurrence compared to $1,820 for nonrecurrence.	Inpatient costs	Supported by Merck & Co., Inc.
Tanaka	201 8	Japan	Systemic lupus erythematosus	Health payer	3 years	295	Direct medical costs including inpatient, outpatient, pharmaceuti cals	The mean total all-cause direct medical cost per patient was $29,135 and the corresponding costs increased with SLE severity: mild ($7,184),	Inpatient costs were driven by management (55.6%), followed by medication (17.3%) and operation services (10.1%)	Funded by GlaxoSmithKlin e

								moderate ($16,862), and severe ($46,122) (p < 0.001)		
Tanzer	2013	USA	Systemic lupus erythematosus with and without kidney involvement	Patients	1 year	4,193 SLE patients with kidney involvement	Inpatient costs only	Average hospitalization charge was greater for SLE patients with kidney involvement compared to those without kidney involvement ($43,100 versus $28,500; p < 0.0001).	SLE-associated acute kidney failure, transplant, and end-stage kidney disease	No funding source reported
Ungprasert	2020	USA	Polyarteritis nodosa (PAN)	Health payer	NA	4,110 (61% female)	Direct medical costs including inpatient, outpatient, pharmaceuticals	The mean hospital costs for patients in the PAN cohort was $24,048 compared to $10,887 in the non-PAN cohort	Not reported	No funding source reported

Vogelzang	2017	USA	Renal cell carcinoma	Health payer	1 year	1,711 patients: 526 (41.6% female) initiating pazopanib, 1,185 (42.4% female) initiating sunitinib	Direct medical costs including inpatient, outpatient, pharmaceuticals	Among PAN patients, initiating pazopanib was associated with lower total all-cause health care costs ($8,527 vs. $10,924, respectively [mean difference=$2,397]); total medical costs ($3,991 vs. $5,881); and inpatient costs ($2,040 vs. $3,731; all P<0.01) compared with sunitinib	Not reported	Funded by Novartis
Wang	2023	China	Systemic lupus erythematosus	Patients	18 months	1,778 (92.5% female)	Direct medical costs and non-medical costs	The average annual direct cost per patient was estimated at CNY 29,727	Direct medical costs accounted for 86% of total costs	Funded by CAMS Innovation Fund for Medical Sciences
Yang	2021	USA	Neurofibromatosis type 1 related plexiform neurofibromas	Health payer	3 years	383 (48% female)	Direct medical costs including inpatient, outpatient, pharmaceuticals	Mean (SD) total costs per patient per year during follow-up were $17,275 ($61,903)	Medical costs constituted 85% of the total and were driven by inpatient costs (46% of medical costs)	Funded by Merck & Co., Inc

Zhou	2018	USA	Systemic sclerosis	Health payer	12 months	2,192 (84.3% female) patients and 2,192 matched controls	Direct medical costs and indirect costs including absenteeism and disability costs	Unadjusted total annual direct healthcare costs were significantly higher for patients with Systemic sclerosis ($22,016) than matched controls ($5977). Unadjusted indirect costs were $5170 for patients with Systemic sclerosis versus $1663 for controls.	Of total annual direct costs, $16,269 were medical costs and $5746 pharmacy costs. Of indirect costs, disability accounted for $1,554 and medically related absenteeism $3,616.	No funding source reported

Table S3 – Overview of economic evaluation studies

Study ID	Country	Study design	Disease	Perspective	Time horizon (Discount rate)	Intervention	Costs Driver	Primary result – considered cost effective? (Cost effectiveness, budget impacts, cost analyses)	Funding
Pharmaceutical interventions									
Ambavane 2020	US	Cost-effectivenes s	Renal Cell Carcinoma	Patients	40 years (3%)	nivolumab + ipilimumab vs tyrosine kinase inhibitor (TKI)-initiated sequences	drug acquisition, administration, AE management, disease management	Yes- 3.6-5.3 average QALYs gained for nivolumab + ipilimumab vs 2.1–3.7 QALYs for TKI. Incremental cost per QALY below $150,000 for nivolumab + ipilimumab	Funded by Bristol-Myers Squibb
Amdahl 2016	Canada	Cost-effectivenes s	Renal Cell Carcinoma	Third payer/health system/governme nt	5 years (5%)	pazopanib compared with sunitinib as first-line treatment for patients with mRCC	Drug cost for pazopanib and sunitinib, dispensing and administration, routine follow-up care, disease progression and terminal care, and other direct medical costs	Yes- Pazopanib was estimated to yield 0.059 QALY	Funded by GSK and Novartis
Amdahl 2017	UK	Cost-effectivenes s	Renal Cell Carcinoma	Third payer/health system/governme nt	5 years (3.55%)	pazopanib versus sunitinib	initiation, medication, and dispensing for pazopanib and sunitinib, pre-progression follow-up and monitoring,	Yes- pazopanib was estimated to provide more QALYs (0.0565, 95% CI:−0.0920 to 0.2126) at a lower cost (−£1,061, 95%	not specified

							other mRCC-related care associated with pazopanib and sunitinib treatment during PFS, and post-progression supportive care	CI: −£4,328 to £2,067) versus sunitinib.	
Bensimon 2020	US	Cost-effectivenes s	Renal Cell Carcinoma	Third payer/health system/governme nt	lifetime time horizon (3%)	pembrolizumab/ axitinib versus other first-line treatments of advanced RCC	First line treatment cost, subsequent line treatment cost, AE cost, disease management cost, terminal care cost	Yes- ICER of $95,725/QALY versus sunitinib; $128,210/QALY versus pazopanib; and was dominant versus avelumab/axitinib	not specified
Çakar 2023	Switzerland	Cost-effectivenes s	Renal Cell Carcinoma	Health system	40 years (3%)	nivolumab plus ipilimumab versus both sunitinib and pazopanib for the treatment of frst-line aRCC	Drug acquisition, drug administration, treatment initiation, Disease management, Subsequent treatment costs, terminal care and AE costs	Yes- ICER of CHF108,326/QA LY gained versus sunitinib, and CHF106,996/QA LY gained versus pazopanib	Funded by Bristol Myers Squibb
Capri 2020	Italy	Cost-effectivenes s	Renal Cell Carcinoma	health system	5 years (3%)	pazopanib versus sunitinib as a first-line treatment	Post-progression costs, treatment initiation costs.	Yes- pazopanib had higher QALYs (0.060) at lower costs (-€5,857) versus sunitinib	Funded by Novartis Pharmaceu tical Corporation
Chan 2022	China	Cost-effectivenes s	Metastatic, clear-cell, renal cell carcinoma	Third payer/health system/governme nt and societal	20-year lifetime horizon (3%)	nivolumab-ipilimumab vs pembrolizumab-axitinib vs sunitinib	Costs for drugs, adverse events (AE), disease progression, palliative care, and hospitalization costs	Yes- ICER of $34,190/QALY for nivolumab-ipilimumab vs sunitinib; ICER of pembrolizumab-axitinib dominated by nivolumab-ipilimumab, and	not specified

								$12,630,828/QALY vs sunitinib	
Chen 2019	China	Cost-effectiveness	Renal Cell Carcinoma	Third payer/health system/government and societal	lifetime time horizon (not specified)	pembrolizumab plus axitinib versus sunitinib for previously untreated patients with aRCC	Drug cost, PA subsequent treatment cost, Sunitinib subsequent treatment cost,BSC cost,Terminal care cost, AEs cost,	No- ICER of US$55,185/QALY versus sunitinib	National Natural Science Foundation of China
De Groot 2017	Holland	Cost-effectiveness	Metastatic Renal Cell Carcinoma (mRCC)	Dutch healthcare sector	Lifetime (4%)	targeted therapies vs not receiving targeted therapy at all	not specified	Yes- ICER of €105,011 per QALY gained compared to not using targeted therapy at all	Funded by Pfizer and Roche
Delea 2015	US	Cost-effectiveness	Renal Cell Carcinoma	Health system	37.5 months (3%)	pazopanib versus sunitinib	Medication costs, administration/dispensing, other costs associated with pazopanib and sunitinib treatment, Pre-progression routine follow-up and Post progression routine follow-up costs	Yes- ICER of pazopanib was dominant	Funded by GlaxoSmith Kline
Deniz 2019	US	Cost-effectiveness	Advanced renal cell carcinoma (aRCC)	US third-party payer	25 years (3%)	Sunitinib followed by nivolumab & pazopanib followed by nivolumab vs axitinib, pazopanib, everolimus and cabozantinib as second-line treatments	Medication costs and subsequent supportive care	Yes- Incremental costs per LY gained were $49,592, $73,927 and $30,534 for nivolumab versus axitinib, pazopanib and everolimus-containing sequences, respectively.	Funded by Bristol-Myers Squibb

Ding 2021	US	Cost-effectivenes s	Renal Cell Carcinoma	U.S. payers' perspective	lifetime time horizon (3%)	pembrolizumab plus axitinib versus sunitinib in the first-line setting	Drug cost, AE cost	Yes- ICER of $148,676/QALY	Funded by Merck & Co. Inc.
Gupta 2023	India	Cost-effectivenes s	Renal Cell Carcinoma	Patients & health system	lifetime time horizon (3%)	Sunitinib vs pazopanib vs pembrolizumab/ lenvatinib vs nivolumab/ipilim umab	Per cycle cost of drug, Health system cost (Outpatient consultation, Day-care visit), OOPE, Per cycle cost of management of adverse effects	Yes- Sunitinib incurs an average cost of ₹ 143,269 ($1,939 USD) per QALY lived	Roche (Inst), Sanofi (Inst), Johnson & Johnson (Inst), Amgen (Inst), Celltrion (Inst), Oncostem Diagnostics (Inst), Novartis (Inst), AstraZenec a (Inst), Intas (Inst)
Kim 2021	South Korea	Cost-effectivenes s	Renal Cell Carcinoma	societal perspective	30 years (5%)	cabozantinib compared to nivolumab	Treatment cost, Disease management cost, Adverse event cost, Productivity loss, End-of-life cost	Yes- ICUR of $34,445 per QALY	National Research Foundation of Korea
Kim 2021	Australia	Cost-effectivenes s	Renal Cell Carcinoma	Australian healthcare system	Scenario 1: 60 month (5 yrs) Scenario 2: 110 months (5%)	nivolumab versus everolimus	The cost of nivolumab, time horizon and utilities were main drivers	Yes- ICER of $266,871/QALY for scenario 1 (30 months clinical data), and $213,320/QALY for scenario 2 (80 months clinical data with	None

								updated everolimus price)	
Li 2021	US	Cost-effectivenes s	Advanced renal cell carcinoma (aRCC)	Third payer/health system/governme nt	lifetime time horizon (3%)	Lenvatinib plus pembrolizumab, nivolumab plus cabozantinib, nivolumab plus ipilimumab, pembrolizumab plus axitinib, avelumab plus axitinib, and sunitinib monotherapy	Management of Aes cost, drug cost of (Nivolumab,Ipilimu mab ,AxitinibAvelu mab,Sunitinib,Cabo zantinib,Sorafenib), Cost of BSC, Administration cost,	ICERs of $81282/ QALY for pembrolizumab plus axitinib vs sunitinib and $453391/QALY for nivolumabplus-cabozantinib vs pembrolizumab plus axitinib. The rest of the strategies, such as Lenvatinib plus pembrolizumab, nivolumab plus ipilimumab, and avelumab plus axitinib, were dominated.	None
Li 2021	US	Cost-effectivenes s	Renal Cell Carcinoma	Patients	lifetime time horizon (3%)	nivolumab plus cabozantinib compared with those of sunitinib	Drug cost, Management of Aes, Administration cost	No- ICER of $508,987/QALY	Health and Family Planning Commissio n of Hunan Province, Central Universities of Central South University
Liao 2021	US	Cost-effectivenes s	Renal Cell Carcinoma	not specified	lifetime time horizon (3%)	nivolumab plus cabozantinib with those of sunitinib	Drug costs, cost of managing AE, cost of second-line active treatment	Yes- ICER of $863,720 per QALY gained	National Natural Science Foundation of China, West China Hospital,

									Sichuan University
Lin 2023	China	Cost-effectivenes s	Renal Cell Carcinoma	not specified	10 years (5%)	anlotinib as a first-line treatment for mRCC compared to that of sunitinib	Direct medical costs included the following: drugs, laboratory tests, treatment of AEs (grade >3), and outpatient fees.	Yes- ICER of -$9,210,858.93 per LYs and -$354,117.07 per QALYs gained	not specified
Lu 2020	US	Cost-effectivenes s	Advanced renal cell carcinoma (aRCC)	US payer perspective	10 years (3%)	avelumab plus axitinib versus sunitinib in first line treatment for advanced RCC	differences in utilities in PFS and after progression were the most influential factors	No- ICER of $565,232 per QALY	Natural Science Foundation of Guangdong Province, National Natural Science Foundation of China
Mason 2023	USA	Cost-effectivenes s	Renal Cell Carcinoma	Third payer/health system/governme nt	10 years (3%)	pembrolizumab + lenvatinib followed by cabozantinib vs pembrolizumab + axitinib followed by cabozantinib for patients with favourable risk, nivolumab + ipi limumab followed by cabozantinib vs cabozantinib followed by nivolumab for patients with intermediate/po or risk	price for infusion/injection, prices for oral medications, costs for general treatment and monitoring, BSC, and terminal care	Yes- for patients with favourable risks ICER of $117 625 per QALY; In patients with intermediate or poor risk ICER of $4184 per QALY	None
McCrea 2018	USA	Cost-effectivenes s	Renal Cell Carcinoma	US payer perspective	25 years (3%)	nivolumab versus everolimus	Initial treatment cost (Acquisition, administration,	Yes- ICUR of $US51,714 per QALY gained	None

							monitoring), subsequent treatment cost (Acquisition, administration, monitoring), AE costs	and $US44,576 per life-year gained	
Meng 2018	UK	Cost-effectivenes s	Renal Cell Carcinoma	not specified	30 years (3.5%)	cabozantinib vs the standard of care	treatment costs, costs of adverse events, PFS health state costs, progressed health state costs, and terminal care costs.	Yes- ICER versus axitinib and everolimus were 98,967 GBP/QALY and 137,450 GBP/QALY, respectively	Funded by Ipsen Pharm
Mihajlović 2013	Serbia	Cost-effectivenes s	Renal Cell Carcinoma	not specified	lifetime time horizon (3% for cost, 1.5% for health outcomes)	everolimus vs best supportive care	direct costs of drug treatment and other medical interventions.	No- ICER of €86,978 per QALY	not specified
Nazha 2018	Canada	Cost-utility	Metastatic Renal Cell Carcinoma (mRCC)	Canadian Healthcare System	5 years (1.5%)	sunitinib versus pazopanib in frst-line setting	The major cost component (56%) is related to best supportive care (BSC)	Yes- ICUR of $67,227/QALY for sunitinib versus pazopanib	Canadian Center for Applied Research in Cancer Control, Coté-Sharp Family Foundation at the McGill University Health Center
Petrou 2015	Cyprus	Cost-effectivenes s	Renal Cell Carcinoma	Cyprus healthcare system	10 years (3.5%)	ofaxitinib versus sorafenib, for the second-line treatment	Hospitalization, Specialist visit, FBC, U&E, pharmaceutical cost	Yes- ICER of 87,936 euro per QALY	not specified
Petrou 2014	Cyprus	Cost-effectivenes s	Renal Cell Carcinoma	Cyprus healthcare system	10 years (3.5%)	sorafenib as a second line treatment	Cost distribution of general medical and other	No- ICER of €102,059 per QALY	None

							pharmaceutical costs		
Pruis 2019	Singapore	Cost-effectivenes s	Renal Cell Carcinoma	Singapore healthcare system	10 years (3%)	sunitinib versus inter feronalfa	Drug costs, disease management cost, AE cost	No- ICER of SGD191,061 (USD139,757) per QALY gained	None
Raphael 2018	Canada	Cost-utility	Metastatic Renal Cell Carcinoma (mRCC)	Canadian Healthcare System	Lifetime (3%)	nivolumab vs everolimus	Drug costs, wastage, treatment duration	No- $8138/QALM (Quality Adjusted Life Month) gained	not specified
Redig 2019	Sweden	Cost-effectivenes s	Metastatic Renal Cell Carcinoma (mRCC)	Swedish healthcare system	10 years (not specified)	targeted therapies	TT costs	Yes- Costs per LY gained were estimated at $78,656 for the early TT period (2006–2008) and $34,132 (2009–2010) for the later TT period.	Funded by Pfizer
Reinhorn 2019	US	Cost-effectivenes s	Advanced renal cell carcinoma (aRCC)	U.S. payer perspective	10 years (3%	nivolumab and ipilimumab compared with sunitinib for first-line treatment	not specified	Yes- ICER of $125,739/QALY vs sunitinib	None
Sarfaty 2018	US	Cost-effectivenes s	Renal Cell Carcinoma	US payer perspective	10 years (3%)	nivolumab for second-line treatment	Only direct medical costs were considered, including drug, administration, and adverse event (AE) costs.	mixed results- ICER of $146,532/QALY for nivolumab vs everolimus, and $226,197/QALY versus placebo. Limiting the maximal treatment duration of nivolumab to 2 years reduced the ICER to $121,788/QALY versus everolimus.	None

Sharma 2023	not specified	Cost-effectiveness	Renal Cell Carcinoma	Health system	5 years (3%)	Pembrolizumab	not specified	No- ICER of $331,613/QALY gained	None
Shay 2021	US	Cost-effectiveness	Renal Cell Carcinoma	US payer perspective	10 years (3%)	nivolumab + ipilimumab vs pembrolizumab + axitinib vs avelumab + axitinib	price for either pembrolizumab + axitinib or nivolumab + ipilimumab	Yes- ICER of NI versus PA was $47,504.73/QALY; NI versus AA was $96,533.11/QALY; PA versus AA was $113,015.87/QALY	not specified
Vargas 2019	Chile	Cost-effectiveness/ utility	Metastatic Renal Cell Carcinoma (mRCC)	Chilean health care system	10 years (3%)	sunitinib versus pazopanib and best supportive care as first-line treatment	Utility data	No- ICER of $62,327.11/QALY for PA versus BSC and $85,885/QALY for Sunitinib versus Pazopanib	Pfizer Chile
Wan 2017	US/China	Cost-utility	Advanced renal cell carcinoma (aRCC)	US and Chinese health care systems	Lifetime (3%)	nivolumab for the second-line treatment of mRCC	Drug prices	No- $151,676/QALY	National Natural Science Foundation of China
Wan 2019	USA	Cost-effectiveness/ utility	Metastatic Renal Cell Carcinoma (mRCC)	US payer perspective	Lifetime (3%)	nivolumab plus ipilimumab vs sunitinib in the first-line setting	overall survival hazard ratio and patient weight	Yes- $108,363 per QALY	not specified
Wang H 2022	China	Cost-utility	Advanced renal cell carcinoma (aRCC)	Chinese health care system perspective	20 years (5%)	nivolumab + cabozantinib strategy over sunitinib strategy	Drug prices	No- ICER of $292,945 per QALY	None
Wang Y 2022	China	Cost-utility	Advanced renal cell	Chinese health care system perspective	Lifetime (5%)	lenvatinib plus pembrolizumab group vs	Drug prices	No- ICER of 2,657,025 RMB/QALYs	None

			carcinoma (aRCC)			sunitinib group as first-line treatment			
Watson 2020	USA	Cost-utility	Advanced renal cell carcinoma (aRCC)	US health care sector	not specified (3%)	Pembrolizumab followed by axitinib,	Drug prices	No- ICER of $172,532/QALY	None
Wu 2018	US, UK, China	Cost-utility	Advanced renal cell carcinoma (aRCC)	Third party payer	10 years (3%)	nivolumab plus ipilimumab as first-line therapy to the present standard care	The price of sunitinib, nivolumab and ipilimumab	ICERs for nivolumab plus ipilimumab over sunitinib: US $ 85,506 /QALY; UK $ 126,499/QALY; and China $ 4682/QALY	Shanghai Health Commissio n
Zhu J 2020	USA	Cost-utility	Advanced renal cell carcinoma (aRCC)	US payer perspective	20 years (3%)	pembrolizumab plus axitinib treatment vs sunitinib treatment as a first-line treatment	The cost of pembrolizumab	No- ICER of $249K per QALY compared to WTP of $150k/QALY	National Natural Science Foundation of China (grant no. 71704064), the Natural Science Foundation of Guangdong Province, China
Zhu Y 2023	USA	Cost-effectivenes s/ utility	Advanced renal cell carcinoma (aRCC)	US payer perspective	20 years (3%)	lenvatinib plus pembrolizumab) as the first-line treatment	The most influential factor in this model was the cost of pembrolizumab with LP.	Using a WTP threshold of $150,000 per QALY, LP was cost-effective with an ICER of $131,656 per QALY; LE was not cost-effective with an ICER of 201,928 per QALY	None

Clark 2017	US	Cost-effectivenes s	Autosomal dominant polycystic kidney disease (ADPKD)	US healthcare system	30 days (0%)	ACE (Angiotensin-converting enzymes inhibitors) vs ARB (angiotensin II receptor blockers)	total costs of ACE-I and ARB	Yes- ACE-I dominated ARB	None
Erickson 2013	US	Cost-utility	Autosomal dominant polycystic kidney disease (ADPKD)	Societal	Lifetime (3%)	Tolvaptan vs soc	Drug costs, other care costs (smaller than the drug costs)	No- $744 100 per QALY gained compared with standard care	National Institutes of Health and Agency for Healthcare Research and Quality
Rombach 2013	Netherlands	Cost-effectivenes s	Fabry disease	Dutch healthcare system	lifetime time horizon (0%)	enzyme replacement therapy (ERT) compared to standard medical care	Inpatient hospital cost, drug cost, dialysis cost, transplant, Outpatient hospital cost, Productivity loss, psychologist, physician/other	Yes- extra costs per additional year free of end-organ damage and the extra costs per additional QALY range from €5.5 - €7.5 million	Ministry of Health (ZonMW)
van Dussen 2014	The Netherlands	Cost-utility	Gaucher Disease Type 1	Societal	Lifetime (1.5% for effects and 4% for costs)	enzyme replacement therapy (ERT) compared to standard medical care	Healthcare costs	No- ICER of €884,994 per QALY	Ducth Top Institute Pharma project
Jogimahant i 2021	US	cost effectivenes s	Giant Cell Arteritis	Third payer/health system/governme nt	not specified (not specified)	tocilizumab in GCA compared with prednisone alone	Cost of side effects in patients treated with prednisone and tocilizumab (Cost of Class I Severity, Cost of Class II Severity)	Yes- Three out of the 4 group combinations of tocilizumab with prednisone demonstrated a statistically significant (P , 0.05) difference in cost compared with prednisone alone for GCA	not specified

Alsuwayegh 2023	Saudi Arabia	Cost-effectiveness	Systemic Lupus Erythematosus	Third payer/health system/government	1.5 years (not specified)	Belimumab vs soc	Cost of treatment between Belimumab vs soc	Mixed results- The mean difference in cost and SLEDAI-2K score reduction between belimumab versus the SoC were USD 5303.16 [95% CI: USD 2735.61–USD 7802.52] and 3.378 [95% CI: 1.769–6.831], respectively.	Researchers Supporting Project number (RSP2023R16), King Saud University, Riyadh, Saudi Arabia
Bindra 2023	US	Cost-effectiveness	Systemic Lupus Erythematosus	Third payer/health system/government and societal	3 years (not specified)	Acthar Gel (repository corticotropin injection) versus SoC treatment	Not specified	Yes- From a payer perspective Acthar Gel versus SoC resulted in ICER of $133,110/QALY and $94,818/QALY over 2 and 3 years; From a societal perspective, ICER were $70,827 per QALY and $32,525 per QALY over 2 and 3 years, respectively.	Funded by Mallinckrodt Pharmaceuticals
Otten 2022	UK	HTA	Systemic Lupus Erythematosus	UK healthcare System	lifetime time horizon (3.5%)	belimumab with standard treatment	AMS score coefficient for predicting pulmonary organ	Yes- For the intravenous formulation, the company's and	National Institute for Health Research

							damage, the discontinuation rate following 2 years of treatment, and the treatment effect of belimumab at week 52	ERG’s ICER estimates were £12,335 per QALY and £30,278 per QALY respectively; For the subcutaneous formulation, company’s and ERG’s ICER estimates were £8480 per QALY gained and £29,313 per QALY respectively	(NIHR) Health Technology Assessmen t Programme
Specchia 2014	Italy	HTA	Systemic Lupus Erythematosus	Health system	lifetime time horizon (3%)	Belimumab	not specified	Yes- ICER of €32,859 per quality adjusted life year gained	Funded by GlaxoSmith Kline
Vandewalle 2021	Portugal	HTA	X-linked hypophosphat emia	Third payer/health system/governme nt	not specified (not specified)	burosumab versus conventional therapy	not specified	n/a	Kyowa Kirin Internationa l
Non-pharmaceutical interventions									
Donovan 2022	Canada	Cost-effectivenes s	Renal Cell Carcinoma	Health system	5 years (1.5%)	stereotactic body radiotherapy (SBRT) versus radiofrequency ablation (RFA)	Cost of personal radiation therapy, cost of RFA procedure, cost of SBRT, cost of surveillance CT, monthly, cost of metastatic disease, monthly	Yes- SBRT economically dominated RFA with a gain of 4.103 quality-adjusted life years (QALYs) and a cost of $16,097, compared with 3.607 QALYs at a cost of $18,324 for RFA	Funded by Accuray Incorporate d

McGann 2015	Angola	Cost-effectivenes s	Sickle cell anemia	Third payer/health system/governme nt	10 years (3%)	newborn screening (NBS) and treatment program	discounting rate	Yes- cost per HLY gained is $1380-$3565	Funded by Chevron
Rossi 2021	UK	Cost-effectivenes s	Renal Cell Carcinoma	National Health Service perspective	lifetime time horizon (3.5%)	Screening of 60-year-old men	Screening costs (cost of AAA screening ultrasound in the UK), Assessment costs, Management, Annual drug costs.	mixed results- ICER of £18 092/QALY given a prevalence of RCC of 0.34%; ICER of £37 327/ QALY given a prevalence of RCC of 0.16%	Kidney Cancer UK,The Urology Foundation, Renal Cancer Research Fund, CRUK Prevention Fellowship
Thompson 2022	UK	Cost-effectivenes s	Renal Cell Carcinoma	Third payer/health system/governme nt	lifetime time horizon (3.5%)	Annual renal imaging surveillance	Not specified	Yes- Incremental Net Monetary Benefit (INMB): £3522 (95% CI: −£2747 to £7652), Incremental Life Year Gained (LYG): 1.25 (95% CI: 0.30 to 1.86), and Incremental Quality-Adjusted Life Year (QALY): 0.29 (95% CI:0.07 to 0.43), at an additional mean discounted cost of £2185 per patient (95% CI: £430 to £4144)	NIHR Manchester Biomedical Research Centre

Walpole 2021	Australia	Cost-effectivenes s	Renal Cell Carcinoma	Australian healthcare system	Lifetime (3%)	surveillance of BAP1 germline carriers	Probability of diagnosis and assumption that diagnosis leads to full uptake and adherence	Yes- $1,265 USD per life year gained	Australian Government Research Training Program, National Health and Medical Research Council of Australia, Highland Island Enterprise
Flahault 2017	France	Cost-utility	Autosomal dominant polycystic kidney disease (ADPKD)	Health system	Lifetime (not specified)	Systematic screening of ADPKD	Not identified (health system costs included)	Yes- Systematic screening was deemed cost-effective and provides a gain of 0.68 quality-adjusted life years compared to targeted screening	None
Malhorta 2019	USA	Cost-utility	Autosomal dominant polycystic kidney disease (ADPKD)	Societal	not specified (3%)	MR angiography screening and surveillance strategies	not specified	Yes- ICER of $71,525	not specified
Stavrakas 2022	UK	Cost-effectivenes s	eosinophilic granulomatosis with polyangiitis	Not specified	1 year (not specified)	joint management of AAV	treatment decisions (After clinical assessment, 13 patients had changes made to their ENT treatment, 2 had some changes in their immunosuppressio	Yes- £215 for the MDT clinic vs £352 for attendance at individual clinics	None

							n, while 11 had changes in both ENT and Rheumatology treatment)		
Lambe 2018	Ireland	Cost-effectivenes s	Fabry disease	not specified	1 year (not specified)	screening programme for cohort of stroke patients under 70 years of age	not specified	No	Not specified
Hatam 2013	Iran	Cost-utility	galactosemia	health system	1 year (n/a)	neonatal screening program	Mean cost of performing the screening, Cost of early treatment of screened patients, Cost of delayed treatment of unscreened patients	Yes- $19641 savings per patient	Shiraz University of Medical Sciences
Laskin 2013	US	Cost-effectivenes s	Idiopathic Nephrotic Syndrome	not specified	lifetime time horizon (3%)	LTBI screening strategy	TST (Nurse interpretation, test, travel time), Questionnaire, IGRA (interferon release assay), Deaths, NS relapse and NS onset	Mixed results- At an LTBI prevalence of 1.1%, no screening strategy dominated ($2,201; 29.3356 QALYs) targeted screening ($2,218; 29.3356 QALYs) and universal TST ($2,481; 29.3347 QALYs). At a prevalence >10.3 %, targeted screening with a risk-factor questionnaire was the most cost-effective option. Higher	National Center for Research Resources, the Office of the National Coordinator for Health Information Technology , Gilead Sciences Inc, National Institute of Diabetes and Digestive and Kidney Diseases

								than a prevalence of 58.5%, universal TST was preferred.	
Williams 2019	US	Cost-effectivenes s	Systemic Lupus Erythematosus	US health care sector	12 weeks (not specified)	Lupus Self-management (PALS) program	staff salaries, project overhead, supplies and equipment. The largest cost categories per patient were for participant incentives and cell phones.	Yes- There was a savings of $23,417 per individual receiving the intervention with a benefit-cost ratio of 18.13 per patient	National Institutes of Health/Nati onal Center for Advancing Translation al Sciences, the Rheumatol ogy and Immunolog y Multidiscipli nary Clinical Research Center National Institutes of Health/Nati onal Institute of Arthritis and Skin Diseases
Fallah 2016	not specified	Cost-utility	Tuberous Sclerosis Complex	Third party payer	5 years (3%)	resective epilepsy surgery vs vagus nerve stimulator implantation vs ketogenic diet vs addition of a third ASD	Surgery was the most costly intervention, cost-effectiveness driven by this and utility values	The addition of a third ASD ($6600 for a gain of 4.14 QALYs) was the most cost-effective treatment strategy	None

Jousselme 2022	France	Cost-effectivenes s	thrombotic thrombocytope nic purpura	not specified	1 year (n/a)	ADAMTS13 activity (A Disintegrin and Metalloprotease with ThromboSpondi ntype 1 repeats, member 13)	Biological costs, administrative costs, Cost of one kit	Yes- Mean differences between the two alternative diagnostic tests was s11 323.60.	not specified
Kim 2017	US	Cost-effectivenes s	thrombotic thrombocytope nic purpura	not specified	immediate 3-day outcomes (0%)	ADAMTS13 test and/or PLASMIC score	In-house ADAMTS13 assay, Send-out ADAMTS13 assay, TPE, Infection, Catheter thrombosis, Venous thrombosis, Bleeding or major complications	Yes- ICER of in-house ADAMTS13: $49,644 per death avoided	National Institutes of Health
**Overview of included budget impact and cost analyses**									
Aiello 2023	Italy	Budget impact analysis	familial Mediterranean fever	Health system	3 years	Anakinra	drug cost of Anakinra	cumulative savings of €14,121,080 over 3 years	Sobi S.r.l., Milan, Italy,
Cevey 2019	Spain	Budget impact analysis	Systemic Lupus Erythematosus	Health system	3 years	belimumab IV to belimumab SC until reaching approximately 17% of the market share	Treatment cost: (€11,902.80 per patient for belimumab SC, €12,448.28 per patient for belimumab IV during ongoing phase, and 14,363.40 per patient for belimumab IV on loading phase; Administration cost: €3388.72 per	Savings in direct healthcare costs of 6 million euros over the 3 years.	GlaxoSmith Kline

							patient on ongoing phase and €3910.07 per patient on loading phase		
Clarke 2018	USA	Budget impact analysis	Systemic Lupus Erythematosus	Health payer	4 years	diagnosing SLE using MAP (multivariate assay panel)	Incremental cost of MAP vs SDLTs, Average pre-diagnosis cost per patient with SLE/year, Average pre-diagnosis cost per patient without SLE/yea, Average per patient cost for mild, moderate, severe SLE/year. Average cost of true positive, false positive, true negative, false negative	$1,991,152 cost savings	Not specified
Goshua 2020	USA	Budget impact analysis	thrombotic thrombocytopenic purpura	Health payer	between 1 January 2004 and 31 December 2018	Rituximab	inpatient cost (including inpatient medicine bed, professional and technical cost of TPE) and total cost of treatment with 1 rituximab dose	Cost savings of $905 906 for non-relapse TTP; $425 736 for relapse TTP	None
Hutson 2021	USA	Budget impact analysis	Renal Cell Carcinoma	Health payer	October 1, 2013, through March 31, 2018	first-line (1L) tyrosine kinase inhibitors (TKIs) followed by second-line (2L) therapy	delayed disease progression as a result of 1L, 2L	lower total health care costs ($10,342 vs $13,388; P= 0.0347) per patient per month	EMD Serono Inc.
Khattab 2022	Egypt	Budget impact analysis	Systemic Lupus Erythematosus	Health system	between 2018 and 2020	mycophenolate mofetil or cyclophosphamide as induction therapy	Immunosuppressants, lab tests, hospitalization ICU, physician visits	$2339.69 versus $1329.03 for MMF vs CYC, (p <0.001)	None

Kim 2020	USA	Budget impact analysis	Thrombotic microangiopat hy	Health system	6 days	Delay in ADAMTS13 measurement	The delay modelled to impact speed to treatment which reduces mortality and hospital stay	$27 524 to $46 470 (ADAMTS13 result was available on day 1 to day 5); $4155 to $5123 additional cost per day for delay	Not specified
Lobo 2016	USA	Budget impact analysis	Renal Cell Carcinoma	Not stated (seems to be health payer)	5 years	RCC surveillance guidelines	CXR,Chest CT,Abdominal US,Abdominal CT,Abdominal MRI	Low risk cohort: $587 (CUA), $1,076 (AUA), $1,705 (EAU) and $1,768 (NCCN) in 5 years; High risk cohort: $903 (CUA), $2,525 (EAU) and $3,904 (AUA and NCCN) in 5 years	None
Massachi 2020	USA	Budget impact analysis	Acute intermittent porphyria (AIP)	Health payer	Not specified	hemin vs givosiran	Drug cost, NSAIDs, per day, Opioids, per day, IV infusion (CPT 96365), CVAD (CPT 36561), Comprehensive metabolic panels, Urinalyses (CPT 81000), PCP visits (CPT 99213), Specialist visit (CPT 99214), ER visit, Hospitalization	$482,113 annual savings	Recordati Rare Diseases
Muram 2013	USA	Cost comparison	Neurofibromat osis type 1	Health payer	not specified	genetic testing (SPRED1 testing alone vs NF1 mutation analysis with	Genetics exam, Ophthalmology exam, Magnetic Resonance Imaging, NF1	$14-$16 per individual	Not specified

						reflex to SPRED1)	mutation analysis, SPRED1 mutation analysis, Reflex SPRED1 mutation analysis		
Nalysnyk 2018	USA	Budget impact analysis	Gaucher disease type 1	Health payer	3 years	GDT1 Eliglustat	Administration cost by site of care (home, outpatient, hospital), Drug acquisition costs (Imiglucerase, Velaglucerase, Taliglucerase, Eliglustat, Miglustat)	$1,526,710 savings annually	Sanofi Genzyme
Perrin 2015	USA	Cost comparison	Renal Cell Carcinoma	Health payer	10.6 years	everolimus vs axitinib	drug acquisition costs, general practitioner and nurse visits, imaging and lab tests, hospitalizations, adverse events, post-progression therapies, and palliative care.	$12,985 (lifetime horizon)	Novartis Pharmaceuticals Corporation
Petrou 2023	Cyprus	Budget impact analysis	Amyloid light chain (AL) amyloidosis	Health system	5 years	Daratumumab	drug cost, other medication cost, administration and monitoring cost, AE cost	€254,264 (1st year) - €497,007 (5th year)	None
Pierotti 2016	Italy	Budget impact analysis	Systemic Lupus Erythematosus	Health system	4 years	Belimumab	Belimumab, NSAIDs, Corticosteroids, Antimalarials, Immunosuppressants, Biologics	€6.2 million (3 years)	GlaxoSmith Kline
Pollissard 2021	USA	Budget impact analysis	Thrombotic thrombocytopenic purpura	Health payer	not specified	Caplacizumab	Hospitalization (General ward day, ICU day, TPE procedure), Caplacizumab one	$14,000	Sanofi

							dose, Therapeutic plasma exchange.		
Racsa 2015	USA	Budget impact analysis	Renal Cell Carcinoma	Health payer	Between 1 November 2009 and 31 December 2012	sunitinib vs pazopanib	First year medical cost, Index medication, non-index medication, RCC related, non-index, non-RCC related, adherent cost	total healthcare costs trended higher with sunitinib with mean difference of $12,192	Comprehensive Health Insights, Humana Inc.
Swallow 2018	USA	Cost comparison	Metastatic Renal Cell Carcinoma (mRCC)	Health payer	1- and 2-year horizon	cabozantinib, nivolumab, and axitinib with everolimus for the second-line	price of the drugs, and the duration of treatment.	Additional costs of treatment with cabozantinib, nivolumab, or axitinib were $34,141, $19,371, and $17,506 higher, respectively.	Novartis Pharmaceuticals
Vogelzang 2018	USA	Budget impact analysis	Advanced renal cell carcinoma (aRCC)	Health payer	Not specified	pazopanib vs sunitinib	Inpatient, outpatient, ER costs	$2,397 total healthcare cost; $1,890 medical cost; $1,691 inpatient cost	Novartis Pharmaceuticals
White 2021	USA, UK, France	Cost savings	Thrombotic thrombocytopenic purpura	Health payer	Not specified	ADAMTS-13 activity assay	incidence of TMA, cost of one TPE treatment, cost per HemosIL AcuStar ADAMTS-13 Activity test, Cost per standard ADAMTS-13 activity test	$18 million, £1.2 million, and €1.6 million annually in US, UK, France	Instrumentation Laboratory
Villa 2013	Spain	Budget impact analysis	Renal Cell Carcinoma	Health system	3 years	Pazopanib	Pharmacological costs per cycle for pazopanib and sunitinib, cost associated with the management of AE were	€6,723,622 savings	GlaxoSmith Kline España

Gil-Rojas 2022	Colombia	Cost savings	Thrombotic thrombocytopenic purpura	Health payer	1 year	Early identification and treatment of TTP	Diagnostics process cost, support measures, treatment cost, hospitalization	Estimated cost per avoided death was $3414	Sanofi Colombia
Botrel 2021	Brazil	Cost analysis	Renal Cell Carcinoma	Health payer	12 months	pembrolizumab plus axitinib and nivolumab plus ipilimumab	Drugs and monitoring costs	P + A suggests better economic scenario versus N + I	Merck Sharp & Dohme
MacLean 2016	USA	Cost comparison	Advanced renal cell carcinoma (aRCC)	Health payer	2009-2013	sunitinib and pazopanib	NA	daily mean index medication costs were: sunitinib ($216) vs pazopanib ($177)	Pfizer
Shi 2018	China	Cost analysis	Metastatic Renal Cell Carcinoma (mRCC)	Health payer	Not specified	Sequential treatment regimens for mRCC	Medication costs, overall AE costs	cost per patient per treatment month lowest for sunitinib + axitinib (¥14,898), highest for sunitinib + sorafenib (¥20,103)	Pfizer
Shenavandeh 2021	Iran	Cost analysis	Systemic sclerosis	Health payer	Not specified	local injection of botulinum toxin-A (BTX-A) and intravenous prostaglandin analogs (iloprost/alprostadil) in patients with SSc with resistant digital ulcers	Medication costs, overall AE costs	The cost was significantly lower in the BTX-A injection group ($p < 0.0001$)	Shiraz University of Medical Sciences
Shih 2019	USA	Cost analysis	Renal Cell Carcinoma	Health payer	2007-2011	new oncologic technologies	NA	new cancer drugs key drive of economic burden	China Medical University Hospital, National Cancer Institute,
Vuorinen 2019	Finland	Cost analysis	Metastatic Renal Cell	Health system	4 years duration	Sunitinib	Drug prices	Total treatment cost was 30,530 €/patient.	Pfizer Finland, Research,

			Carcinoma (mRCC)						Development and Innovation Center of Tampere University hospital
